# Excess risks of long COVID symptoms compared with identical symptoms in the general population: A systematic review and meta-analysis of studies with control groups

**DOI:** 10.7189/jogh.14.05022

**Published:** 2024-08-12

**Authors:** Zijun Xu, Wenyue Wang, Dexing Zhang, King Wa Tam, Yiqi Li, Dicken Cheong Chun Chan, Zuyao Yang, Samuel Yeung Shan Wong

**Affiliations:** JC School of Public Health and Primary Care, The Chinese University of Hong Kong, Hong Kong, China

## Abstract

**Background:**

It is important to understand the excess risks of symptoms of long COVID when compared to the same symptoms in the general population. We aimed to evaluate the association between coronavirus disease 2019 (COVID-19) infection and various long-term symptoms.

**Methods:**

We conducted a systematic review and meta-analysis of studies measuring long COVID symptoms lasting for at least three months after severe acute respiratory syndrome coronavirus 2 (SARS-CoV-2) infection in comparison to non-COVID-19 control groups. We searched MEDLINE and Embase (via Ovid), CINAHL (via EBSCOhost), the ProQuest Coronavirus Research Database, and the World Health Organization COVID-19 Research Database for relevant literature on 14 February 2023. The symptom list had 10 categories with 29 symptoms, including general, neurologic, respiratory, cardiac, dermatologic, eye, ear, musculoskeletal, psychiatric, and gastrointestinal symptoms. We performed random-effects meta-analysis and summarised the results using odds ratios (OR) and 95% confidence intervals (CI), after which we conducted subgroup analyses.

**Results:**

We included 51 studies with 17 901 204 participants (range of mean age: 5.9–65.4 years; range of proportion of women: 11.2–96.0%). In the primary analysis, participants with COVID-19 had a significantly higher risk of having at least one long COVID symptom (OR = 2.032; 95% CI = 1.787–2.310). Specifically, they had higher risks of 25 symptoms, the highest of which were for smell (OR = 8.474; 95% CI = 6.357–11.295), taste (OR = 5.881; 95% CI = 3.818–9.059), post-exertional malaise (OR = 3.187; 95% CI = 2.602–3.904), shortness of breath (OR = 2.497; 95% CI = 2.125–2.935), brain fog (OR = 2.093; 95% CI = 1.362–3.218), hair loss (OR = 2.082; 95% CI = 1.291–3.358), chest pain (OR = 2.056; 95% CI = 1.692–2.498), cognitive decline (OR = 1.992; 95% CI = 1.560–2.544), palpitations (OR = 1.986; 95% CI = 1.647–2.395), and fatigue (OR = 1.971; 95% CI = 1.781–2.182). We found significant differences between studies with different follow-up times in cognitive decline, dizziness, palpitations, and sleep problems (*P* < 0.05). Adults had significantly higher risks of cognitive decline, hair loss, and joint pain than children (*P* < 0.05).

**Conclusions:**

We found that COVID-19 can significantly increase the risk of many long COVID symptoms, without differences due to gender, age, or decrease over time after three months post-infection. This highlights that services and interventions for long COVID symptoms are needed.

**Registration:**

PROSPERO (CRD42023409847).

As of 21 September 2023, more than 770 million confirmed coronavirus disease 2019 (COVID-19) cases worldwide have been reported to the World Health Organization (WHO), accounting for about 10% of the global population [1]. The true number of cases, however, may even be greater. These individuals who have been infected may be further affected by COVID-19 in the long term, a condition known as ‘long COVID’. According to the National Institute for Health and Care Excellence (NICE), post-COVID-19 syndrome, also called long COVID symptoms, are signs and symptoms that develop during or after an infection consistent with COVID-19, continue for more than 12 weeks, and are not explained by an alternative diagnosis [2]. The WHO, in turn, defined the condition as the continuation or development of new symptoms three months after the initial severe acute respiratory syndrome coronavirus 2 (SARS-CoV-2) infection, with these symptoms lasting for at least two months and having no other possible explanation [3]. These symptoms can impact the quality of life of affected individuals, resulting in a great burden of disability at the individual and population level [4,5]. This makes understanding the risk of long COVID symptoms crucial to improving their future management and treatment.

Existing systematic reviews have summarised the epidemiology of long COVID symptoms. For example, one meta-analysis of 14 cohort studies found that the most common symptoms were hair loss (24.76%), prolonged palpitation (19.38%), and sleep difficulty (17.87%) [6]. Another synthesis of 38 cohort and cross-sectional studies saw fatigue (48%) and sleep disturbance (44%) as the most common symptoms in chronic post-COVID syndrome [7]. Two systematic reviews reported the prevalence of long COVID symptoms at 12-month follow-up or longer, with one including four studies [8] and another including 18 studies [9].

However, these reviews only reported on the prevalence of symptoms rather than excess risk. They also did not consider comparisons with control groups, with only one meta-analysis included six controlled studies conducted in people 19 years or younger [10]. Such a non-COVID-19 control group could provide information on pre-existing symptom prevalence in the population, in which some symptoms might be caused by existing conditions or seasonal illnesses [4]. Lastly, the follow-up periods in the included studies were short, while the risks in different age groups and gender, as well as the change over time, were also less explored. To address these gaps, we aimed to evaluate the excess risks of long COVID symptoms at least three months after COVID-19 infection in comparison with non-COVID-19 populations.

## METHODS

This was a systematic review and meta-analysis of studies measuring long COVID symptoms in comparison to non-COVID-19 control groups. We registered our protocol in PROSPERO (CRD42023409847) and reported our findings per the Preferred Reporting Items for Systematic Reviews and Meta-Analyses (PRISMA) statement [11].

### Inclusion and exclusion criteria

We included cross-sectional, cohort, and case-control studies with a COVID-19 group and a non-COVID-19 control group. The former consisted of participants with laboratory-confirmed, clinically diagnosed, or rapid antigen test-reported COVID-19, while the latter included participants without confirmed COVID-19 infection, including those untested and tested negative, after the outbreak of COVID-19. Long COVID symptoms had to be categorical outcomes assessed at least three months after disease onset. We set no restriction on age, gender, chronic conditions, or similar characteristics.

We excluded studies with non-COVID-19 control group assessed before the outbreak of COVID-19 to reduce the influence of time, as well as studies with less than 100 COVID-19 participants or with outcomes that report an average level of a scale (e.g. Patient Health Questionnaire-9 for depressive symptoms) without categorical outcomes, as studies with small samples are more likely to be biased or heterogeneous [12,13].

### Search strategy and study selection

We systematically searched MEDLINE, Embase, CINAHL, the ProQuest Coronavirus Research Database, and the WHO COVID-19 Research Database on 14 February 2023 using keywords such as ‘long COVID’, ‘post-COVID syndrome’, names of the specific symptoms, and ‘control’ (Material S1 in the **Online Supplementary Document**). The searches were restricted to humans, English language studies, and publication dates on or after 1 January 2020.

We managed the retrieved records using the Covidence platform. After automatic and manual deduplication, two authors (ZX and WW) screened the titles and abstracts of each record independently using pre-determined criteria, followed by the full texts of potentially relevant studies. The discrepancies at these two stages were resolved by discussion with a third author (DZ).

### Data extraction and risk-of-bias assessment

Two authors (ZX and WW) extracted the data independently into a pre-designed Excel form, again resolving any discrepancies by consultation with a third author (DZ). Another independent reviewer (KWT) randomly checked 10% of the extracted data. The extracted data included study design, country, sample size, participant characteristics, follow-up time, follow-up mode, control condition, and outcome (symptom and effect size). The effect size included crude and adjusted odds ratios (OR), risk ratios (RR), and hazard ratios (HR). We developed the symptom list based on a published paper [14], supplemented by other materials where appropriate. There were 10 categories and 29 symptoms, including neurologic (smell, taste, brain fog, cognitive decline, dizziness, headache, neurological problems), general (post-exertional malaise, fatigue, swelling of legs, fever, sweats, or chills), respiratory (shortness of breath, cough, throat pain), cardiac (chest pain, palpitations), dermatologic (hair loss, skin rash), eye (vision, itchy eyes), ear (hearing), musculoskeletal (muscle pain, joint pain, back pain), psychiatric (sleep problems, depression, anxiety), and gastrointestinal (gastrointestinal symptoms, abdominal pain) symptoms. We defined symptom categories based on the aforementioned published paper and the Common Terminology Criteria for Adverse Events (CTCAE) [14]. As different studies might refer to the same symptom in different ways, we summarised these and presented them in Material S2 in the **Online Supplementary Document**.

Two authors (ZX and WW) assessed the risk of bias of each study independently using an adjusted Newcastle-Ottawa Scale (NOS) (Material S3 in the **Online Supplementary Document**) based on the following factors: representativeness of the COVID-19 participants (sample); selection of the non-COVID-19 participants (control); ascertainment of COVID-19 infection; definition of non-COVID-19 participants; comparability of COVID-19 and non-COVID-19 participants based on the design or analysis; comparability of COVID-19 and non-COVID-19 participants based on the design or analysis; assessment of long COVID symptoms; same method of ascertainment for COVID-19 positive and negative participants; and non-response rate or rate of loss to follow-up. Studies with NOS scores of 7–9, 4–6, and <4 were classified as having a low, moderate, or high risk of bias, respectively [15,16]. Any discrepancies were resolved by discussing with a third author (DZ).

### Statistical analysis

We treated multiple reports on the same study (e.g. one report on three-month outcomes and another on six-month outcomes) as a single study in our analysis. If two reports on the same study reported the same symptoms at the same follow-up time, we included the report with a larger sample size. If the effect sizes of the individual studies were not available, we calculated crude ORs for different symptoms from the numbers of COVID-19 or non-COVID-19 participants with or without specific symptoms. If these numbers were not reported, we calculated them based on the reported prevalence. To assess the long-term effects of COVID-19 infection and reduce the impact of confounders, we used the maximally adjusted effect sizes at the longest follow-up time point for each study in the analysis.

We summarised the results of excess risks for various symptoms as ORs, risk differences (RDs), and their corresponding 95% confidence intervals (CI). We then used random-effects meta-analysis to calculate the pooled OR and 95% CIs for different symptoms and to pool the prevalence of symptoms in non-COVID-19 participants (P0), summarised as percentages and 95% CIs. We calculated RRs and 95% CIs from the formula RR = OR/((1 − P0) + (P0 × OR)) and the RDs with their 95% CIs using the formula RD = P0 × (RR − 1).

We performed subgroup analyses by follow-up duration (3–6, 6–12, and ≥12 months), age (<18 and ≥18 years old), gender (women and men), risk of bias (low and moderate), study design (cohort and cross-sectional study), and adjustment (crude and maximally adjusted), which we had categorised based on the extracted data. We also conducted sensitivity analyses using the results at the shortest follow-up time point of each study. The results of pooled overall risks were visually presented in forest plots. Lastyly, we used *I*^2^ statistics and τ^2^ values to assess heterogeneity [17], with *I*^2^ values of 25%, 50%, and 75% and τ^2^ values of 0.04, 0.08, and 0.16 suggesting low, moderate, and high heterogeneity, respectively [18]. We otherwise assessed for publication bias using Egger’s test [19].

We considered a *P*-value <0.05 as statistically significant. All analyses were done in Stata, version 16.0 (StataCorp LLC, College Station, TX, USA).

## RESULTS

### Study selection, baseline characteristics, and quality assessment

**Figure 1:**
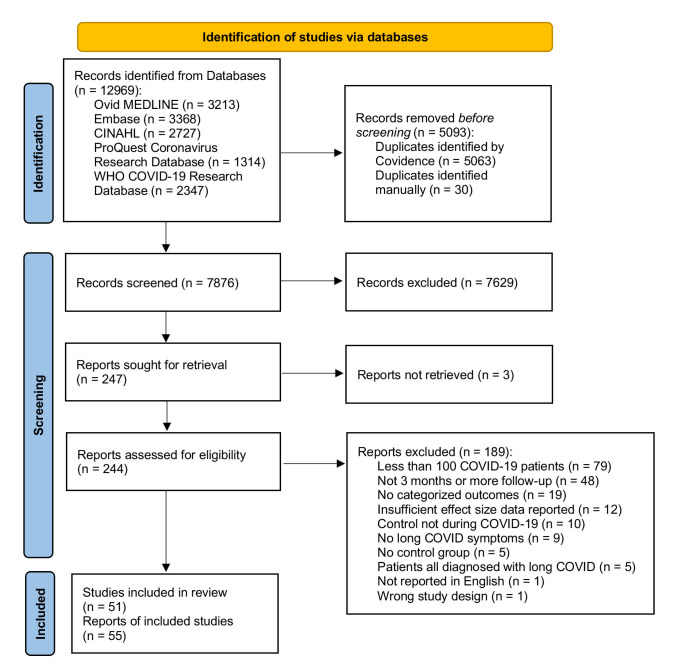
PRISMA flow diagram of the systematic review.

We retrieved 12 969 records from the five databases and removed 5093 duplicates. After screening the titles and abstracts of 7876 records, we reviewed the full texts of 244. Among them, 55 reported the results of 51 studies and were included in the analysis (**Figure 1**).

The 51 studies included 1 213 475 COVID-19 participants (range of mean age: 5.9–65.4 years; range of proportion of women: 11.2%–83.0%) and 16 687 729 non-COVID-19 participants (range of mean age: 7.8–62.8 years; range of proportion of women: 11.0%–96.0%) (Table S1 in the **Online Supplementary Document**), with sample sizes ranging from 107 to 486 149 for the former and from 37 to 8 330 986 for the latter group. Thirty-three (64.7%) studies mentioned using polymerase chain reaction (PCR) to confirm COVID-19. In terms of design, there were 30 prospective cohorts, 14 retrospective cohorts, and 7 cross-sectional studies. The baseline report [20] of Spiliopoulos et al.’s study [21] was defined as a cross-sectional study. The follow-up time ranged from 3 to 24 months. Regarding follow-up mode, 12 studies used health or medical records and 38 studies used questionnaires, which were conducted entirely online in 20 studies and entirely by telephone in 6 studies.

Thirty-three (64.7%) of the 51 studies had a low risk of bias, and the remaining 18 (35.3%) studies had a moderate risk of bias (Table S2 in the **Online Supplementary Document**). Most of the included studies had low risks of bias in the representativeness of COVID-19 participants (n = 33, 64.7%), selection of non-COVID-19 participants (n = 47, 92.2%), ascertainment of infection (n = 49, 96.1%), definition of non-COVID-19 (n = 43, 84.3%), comparability (n = 33, 64.7% for most important factors; n = 30, 58.8% for additional factors), assessment of symptoms (n = 50, 98.0%), same ascertainment method (n = 51, 100%), and sufficient responses or follow-up (n = 38, 74.5%).

### Meta-analysis of the risk of long COVID symptoms

**Figure 2:**
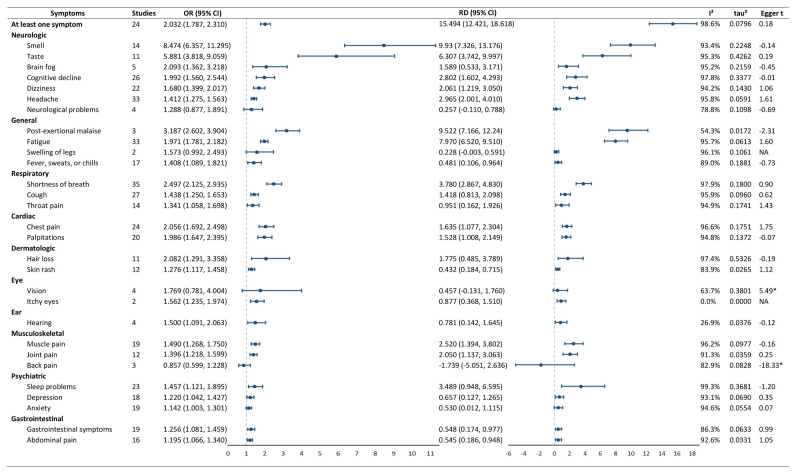
Forest plot of the pooled risks for different symptoms. *P* < 0.05. CI – confidence interval, OR – odds ratio, RD – risk difference.

In the primary analysis (**Figure 2**), COVID-19 participants had a significantly higher risk of having at least one long COVID symptom than non-COVID-19 participants (OR = 2.032; 95% CI = 1.787–2.310, *I*^2^ = 98.6%, τ^2^ = 0.0796). Specifically, COVID-19 participants had significantly higher risks of 25 long COVID symptoms out of 29 symptoms, with the highest risk observed for smell (OR = 8.474; 95% CI = 6.357–11.295, *I*^2^ = 93.4%, τ^2^ = 0.2248), taste (OR = 5.881; 95% CI = 3.818–9.059, *I*^2^ = 95.3%, τ^2^ = 0.4262), post-exertional malaise (OR = 3.187; 95% CI = 2.602–3.904, *I*^2^ = 54.3%, τ^2^ = 0.0172), shortness of breath (OR = 2.497; 95% CI = 2.125–2.935, *I*^2^ = 97.9%, τ^2^ = 0.1800), brain fog (OR = 2.093; 95% CI = 1.362–3.218, *I*^2^ = 95.2%, τ^2^ = 0.2159), hair loss (OR = 2.082; 95% CI = 1.291–3.358, *I*^2^ = 97.4%, τ^2^ = 0.5326), chest pain (OR = 2.056; 95% CI = 1.692–2.498, *I*^2^ = 96.6%, τ^2^ = 0.1751), cognitive decline (OR = 1.992; 95% CI = 1.560–2.544, *I*^2^ = 97.8%, τ^2^ = 0.3377), palpitations (OR = 1.986; 95% CI = 1.647–2.395, *I*^2^ = 94.8%, τ^2^ = 0.1372), and fatigue (OR = 1.971; 95% CI = 1.781–2.182, *I*^2^ = 95.7%, τ^2^ = 0.0613) (**Figure 2**; Figure S1 in the **Online Supplementary Document**). Overall, 40.9% of the participants in the included studies reported long COVID-19 symptoms (Table S3 in the **Online Supplementary Document**). The 10 most prevalent symptoms were fatigue (18.0%), post-exertional malaise (14.6%), sleep problems (12.2%), back pain (12.1%), smell problems (11.4%), headache (11.1%), muscle pain (8.1%), taste problem (7.7%), joint pain (7.7%), and shortness of breath (6.5%).

The results remained stable in our sensitivity analysis using the data at the shortest follow-up time point of each study (Table S4 in the **Online Supplementary Document**).

### Subgroup analysis

We observed significand differences between studies with different follow-up times in four symptoms: cognitive decline (3–6 months: OR = 1.364, 95% CI = 1.039–1.792; 6–12 months: OR = 2.794, 95% CI = 1.391–5.611; ≥12 months: OR = 2.895, 95% CI = 1.773–4.727), dizziness (3–6 months: OR = 1.394, 95% CI = 1.155–1.682; 6–12 months: OR = 2.005, 95% CI = 1.525–2.636; ≥12 months: OR = 2.249, CI = 1.961–2.578), palpitations (3–6 months: OR = 1.722, CI = 1.415–2.094; 6–12 months: OR = 1.826, CI = 1.330–2.506; ≥12 months: OR = 3.587, CI = 2.718–4.733), and sleep problems (3–6 months: OR = 1.156, 95% CI = 1.016-1.316; 6–12 months: OR = 1.385, 95% CI = 0.978–1.960; ≥12 months: OR = 2.272, 95% CI = 1.496–3.450) (**Table 1**).

**Table 1:** Subgroup analysis by different follow-up periods

	3–6 months				6–12 months				12 months or more		
**Symptoms**	**Number of studies**	**OR (95% CI)**	***I*^2^ (%)**	**Number of studies**		**OR (95% CI)**	***I*^2^ (%)**	**Number of studies**		**OR (95% CI)**	***I*^2^ (%)**
At least one symptom	9	2.020 (1.779– 2.294)	94.9	11		2.502 (1.985–3.155)	93.1	5		1.548 (0.629–3.813)	97.3
Neurologic											
*Smell*	10	7.648 (4.505–12.986)	97.4	5		14.945 (10.211–21.875)	78.7	4		9.292 (4.978–17.344)	69.9
*Taste*	6	4.877 (2.275–10.454)	97.5	4		8.488 (2.964–24.305)	92.4	3		11.611 (9.553–14.113)	0.0
*Brain fog*	4	1.897 (1.160–3.104)	96.3	1		3.200 (2.117–4.838)	NA	NA		NA	NA
Cognitive decline*	13	1.364 (1.039–1.792)	98.2	14		2.794 (1.391–5.611)	99.1	6		2.895 (1.773–4.727)	86.0
Dizziness*	13	1.394 (1.155–1.682)	94.2	11		2.005 (1.525–2.636)	95.0	4		2.249 (1.961–2.578)	0.0
*Headache*	19	1.436 (1.294–1.593)	94.5	15		1.443 (1.136–1.833)	96.4	7		2.067 (1.403–3.044)	84.3
General											
*Post-exertional malaise*	2	3.282 (2.606–4.132)	68.3	1		3.140 (2.140–4.607)	NA	1		2.640 (1.650–4.224)	NA
*Fatigue*	21	1.713 (1.506–1.949)	96.5	15		2.560 (1.895–3.459)	96.9	7		1.913 (1.055–3.469)	94.7
*Fever, sweats, or chills*	12	1.583 (1.226–2.042)	90.3	7		1.003 (0.731–1.376)	48.6	2		1.157 (0.966–1.385)	0.0
Respiratory											
*Shortness of breath*	19	2.347 (1.964–2.805)	97.4	18		3.056 (2.076–4.499)	98.0	8		2.465 (1.133–5.362)	96.5
*Cough*	14	1.379 (1.127–1.686)	96.9	13		1.465 (1.237–1.735)	86.6	4		1.798 (0.992–3.259)	90.5
*Throat pain*	9	1.444 (1.136–1.835)	94.5	6		1.403 (1.080–1.824)	93.6	2		3.433 (0.277–42.543)	97.9
Cardiac											
*Chest pain*	14	1.724 (1.480–2.008)	89.9	10		2.491 (1.597–3.887)	97.7	5		2.435 (1.409–4.208)	78.6
Palpitations*	11	1.722 (1.415–2.094)	90.9	9		1.826 (1.330–2.506)	80.0	3		3.587 (2.718–4.733)	0.0
Dermatologic											
*Hair loss*	5	2.681 (1.951–3.684)	90.9	6		1.740 (0.907–3.339)	81.2	2		2.382 (1.855–3.059)	0.0
*Skin rash*	5	1.126 (0.981–1.292)	78.1	5		1.127 (0.880–1.443)	43.6	2		4.947 (0.713–34.33)	88.3
Eye											
*Vision*	NA	NA	NA	4		1.929 (0.846–4.397)	72.1	1		1.630 (0.679–3.915)	NA
Ear											
*Hearing*	1	1.520 (0.768–3.010)	NA	2		1.763 (0.573–5.426)	59.9	1		0.740 (0.293–1.866)	NA
Musculoskeletal											
*Muscle pain*	7	1.376 (1.027–1.843)	95.9	9		1.229 (1.019–1.481)	81.6	3		3.572 (0.806–15.831)	94.7
*Joint pain*	5	1.206 (1.124–1.294)	0.0	4		1.136 (0.927–1.391)	48.0	3		1.864 (1.243–2.794)	48.7
*Back pain*	2	0.882 (0.502–1.550)	89.3	1		0.790 (0.595–1.049)	NA	NA		NA	NA
Psychiatric											
Sleep problems*	10	1.156 (1.016–1.316)	89.8	8		1.385 (0.978–1.960)	73.1	4		2.272 (1.496–3.450)	72.1
*Anxiety*	9	0.959 (0.857–1.074)	84.8	6		1.358 (0.932–1.980)	80.0	4		1.416 (0.774–2.589)	84.6
*Depression*	8	1.094 (0.925–1.293)	66.0	7		1.184 (0.968–1.448)	35.0	4		1.024 (0.801–1.308)	0.0
Gastrointestinal											
*Gastrointestinal symptoms*	9	1.433 (1.250–1.644)	75.9	9		1.115 (0.931–1.336)	43.0	4		1.038 (0.622–1.732)	69.4
*Abdominal pain*	8	1.270 (1.125–1.434)	88.7	6		1.100 (0.898–1.348)	88.5	3		0.935 (0.465–1.879)	79.0

CI – confidence interval, NA – not available, OR – odds ratio

*Significant between-group difference with *P* < 0.05.

Adults had significantly higher risks of three symptoms than children, which were cognitive decline (adults: OR = 2.232, 95% CI = 1.608–3.099; children: OR = 1.155, 95% CI = 0.895–1.490), hair loss (adults: OR = 2.071, 95% CI = 1.096–3.915; children: OR = 0.923, 95% CI = 0.718–1.188), and joint pain (adults: OR = 1.372, 95% CI = 1.188–1.586; children: OR = 0.802, 95% CI = 0.627–1.026) (**Table 2**).

**Table 2:** Subgroup analysis by different age groups

	<18 years old			18 years old or above		
**Symptoms**	**Number of studies**	**OR (95% CI)**	***I*^2^ (%)**	**Number of studies**	**OR (95% CI)**	***I*^2^ (%)**
At least one symptom	8	1.989 (1.656–2.390)	82.1	16	1.851 (1.568–2.184)	96.7
Neurologic						
*Smell*	1	11.761 (9.442–14.65)	NA	12	8.490 (5.560–12.965)	97.5
*Taste*	1	6.102 (3.351–11.109)	NA	9	3.230 (1.218–8.564)	98.5
Cognitive decline*	5	1.155 (0.895–1.490)	26.0	20	2.232 (1.608–3.099)	98.1
*Dizziness*	6	1.812 (1.193–2.753)	89.9	15	1.527 (1.190–1.961)	97.2
*Headache*	6	1.534 (1.045–2.251)	94.1	23	1.368 (1.173–1.594)	97.5
*Neurological problems*	1	1.290 (0.558–2.981)	NA	3	1.284 (0.826–1.997)	85.8
General						
*Fatigue*	6	2.026 (1.355–3.031)	82.7	23	1.984 (1.658–2.373)	98.6
*Fever, sweats, or chills*	4	1.815 (0.912–3.612)	89.8	11	1.376 (1.062–1.783)	91.3
Respiratory						
*Shortness of breath*	7	2.481 (1.486–4.141)	96.3	26	2.548 (1.988–3.265)	98.7
*Cough*	6	1.649 (1.057–2.572)	94.6	18	1.341 (1.155–1.557)	96.2
*Throat pain*	4	1.955 (1.200–3.185)	94.3	10	1.149 (0.902–1.465)	95.6
Cardiac						
*Chest pain*	5	1.750 (1.069–2.866)	92.8	19	2.015 (1.573–2.580)	98.1
*Palpitations*	4	1.290 (0.760–2.190)	85.1	16	2.018 (1.643–2.480)	95.6
Dermatologic						
Hair loss*	2	0.923 (0.718–1.188)	0.0	9	2.071 (1.096–3.915)	98.2
*Skin rash*	3	1.010 (0.745–1.369)	58.3	9	1.285 (1.107–1.492)	87.2
Eye						
*Vision*	1	1.000 (0.772–1.295)	NA	4	1.771 (0.756–4.150)	66.0
Musculoskeletal						
*Muscle pain*	3	1.484 (0.683–3.223)	94.8	14	1.404 (1.131–1.742)	97.1
Joint pain*	1	0.802 (0.627–1.026)	NA	11	1.372 (1.188–1.586)	93.4
Psychiatric						
*Sleep problems*	3	1.098 (0.485–2.488)	43.8	21	1.480 (1.120–1.956)	99.3
*Anxiety*	4	1.148 (0.650–2.029)	74.2	16	1.149 (0.983–1.343)	95.2
*Depression*	3	0.843 (0.405–1.756)	68.2	16	1.217 (1.032–1.434)	93.9
Respiratory						
*Shortness of breath*	7	2.481 (1.486–4.141)	96.3	26	2.548 (1.988–3.265)	98.7
*Cough*	6	1.649 (1.057–2.572)	94.6	18	1.341 (1.155–1.557)	96.2
*Throat pain*	4	1.955 (1.200–3.185)	94.3	10	1.149 (0.902–1.465)	95.6
Gastrointestinal						
*Gastrointestinal symptoms*	4	1.154 (0.945–1.411)	0.0	15	1.253 (1.069–1.468)	89.1
*Abdominal pain*	2	1.000 (0.592–1.689)	96.6	13	1.190 (1.040–1.361)	93.6

CI – confidence interval, NA – not available, OR – odds ratio

*Significant between-group difference with *P* < 0.05.

We saw no significant differences between women and men (*P* > 0.05). Studies with a low risk of bias had significantly lower risks of six symptoms – vision problems, joint pain, anxiety, depression, gastrointestinal symptoms, and abdominal pain – than studies with a moderate risk of bias (*P* < 0.05). The ORs of 13 symptoms in cross-sectional studies were significantly larger than those in cohort studies. Studies with adjusted results had significantly lower risks of at least one symptom, chest pain, anxiety, and depression than studies with crude results (*P* < 0.05) (Tables S5–8 in the **Online Supplementary Document**).

## DISCUSSION

### Key findings

In this systematic review of studies with control groups, we found increased risks of 25 long COVID symptoms at least three months after infection in COVID-19-infected patients, who had more than twice the risk of having a least one long COVID symptom as the non-COVID-19 group. Symptoms with the highest increased risk were smell, taste, post-exertional malaise, shortness of breath, brain fog, hair loss, chest pain, cognitive decline, palpitations, and fatigue. In the subgroup analyses, we observed that risk of most long COVID symptoms may not decrease over time after three months post-infection; that adults had higher risks of cognitive decline, hair loss, and joint pain than children; and that there was no difference between men and women. Lastly, we found more than 40% of COVID patients experienced long COVID symptoms.

Our finding that the risks of most long-term symptoms related to COVID-19 did not decrease over time is consistent with previous studies, although they only reported prevalence instead of risk. A previous meta-analysis [22] found a steep decrease in the prevalence of long COVID symptoms initially in one month, followed by stabilization at approximately 50% during the first year of follow-up. One meta-analysis of 57% of patients reported at least one long COVID symptom at 12 months and above follow-up periods, with the prevalence of different symptoms ranging from 1% to 31% [23], while another found no significant association between follow-up time and the estimated prevalence of one or more long COVID symptoms [24]. A cohort study found people who did not recover within 12 months appeared to develop chronic health conditions and showed little improvement after 12 months [4,25]. Taken as a whole, these findings suggest that COVID-19 has long-term adverse effects.

There were significant differences between adjusted results and crude results for some long COVID symptoms, which suggested there could be other factors associated with our findings. Here we used the maximally adjusted results minimize the effect. According to published research, factors such as female sex, older age, smoking, hospitalisation, chronic conditions, socioeconomic status, and ethnic minority groups were associated with more long COVID symptoms [26–29]. We saw no significant difference between women and men in our systematic review, which might be due to the small number of studies reporting these two subgroups (n/N = 7/51). Moreover, we found adult COVID-19 patients had a higher risk of cognitive decline, hair loss, and joint pain than children. Adults are more likely to have pre-existing health conditions, which can increase the severity of COVID-19 symptoms and the risk of complications [30]. Studies also suggest that features of children’s immune systems might play a role in protecting them from adverse outcomes [31]. Moreover, cross-sectional studies tended to report higher risks of long COVID symptoms than cohort studies. In this systematic review, 50% of cross-sectional studies and 66% of cohort studies had low risks of bias; however, cross-sectional studies had higher risks than cohort studies in regard to both confirmation and comparability of COVID-19 and non-COVID-19 participants. Considering this, the selection and quality of study design for future reviews like ours requires careful consideration.

### Potential mechanisms of long COVID symptoms

Long COVID is a multisystemic condition, which is consistent with the finding that COVID-19 increased the risk of most of the symptoms in our analysis. The underlying pathophysiology of long COVID is still not fully understood, but several potential mechanisms have been proposed, including viral persistence, immune dysregulation, organ dysfunction, disruption in cellular energy production, and abnormalities in tissue oxygen availability. For example, studies have found viral persistence might serve as a driver of long COVID symptoms, with viral proteins and/or RNA found in patients with persistent symptoms after COVID-19 in various organs and tissues [32,33]. Dysregulation of the immune system has also been suggested as a possible mechanism of long COVID [33,34], while some studies have observed that long COVID symptoms may be related to organ dysfunction developed during acute illness [35]. Potential mechanisms for fatigue and other symptoms also include disruption in cellular energy production due to mitochondrial dysfunction, as well as abnormalities in tissue oxygen availability, due to coagulopathy and endothelial damage [34]. However, more research is still needed to fully understand the mechanisms underlying long COVID.

### Current inadequate awareness of long COVID-19 among the public and professionals

There is still a significant gap in awareness and understanding of long COVID among the public. A qualitative study showed that participants were unaware of the common symptoms and risk groups associated with long COVID, even if they had experienced most of the common long COVID symptoms [36]. In another qualitative study, participants who reported persistent or new symptoms more than 12 weeks following COVID-19 were not all familiar with the term ‘long COVID’, with some thinking that COVID-19 was not the actual cause of their symptoms [37]. A Google Trends analysis found that the public interest in long COVID symptoms was significantly lower than for COVID-19, but that there was a sustained increase over time [38]. Conversely, health care professionals recognise long COVID to some extent; for example, a long COVID bulletin from the Public Health Scotland Knowledge Services regularly provides information on the guidelines, epidemiology studies, services, and impacts of long COVID for health care professionals [39]. Still, more work is needed in training health care professionals in raising awareness of long COVID-19 symptoms in clinical practices.

### Current treatment and services for long COVID-19 and the gaps

Current broad diagnostic and treatment options for long COVID symptoms are insufficient [40], with most being pharmacological treatments tested in small-scale pilot studies or treatments effective in other diseases. A previous study suggested that exercise might be harmful for long COVID patients with chronic fatigue syndrome or post-exertional malaise [40]. Other non-pharmacological treatments are very limited and need further development. According to the WHO, countries have integrated COVID-19 services into essential health services, including services for post-COVID-19 conditions [41]. Long COVID clinics have been established in some countries around the world, such as the UK, USA, Belgium, France, Germany, Spain, and Austria [42]. However, there still exist some bottlenecks in terms of long COVID treatment, such as limited manpower, long waiting times, lack of funding, and lack of clear treatment plans [41,42], with clinicians seemingly having less knowledge and training regarding long COVID management [43]. This suggest that there is still a need for professional training to address the gaps in the health systems' readiness for long COVID.

According to the Centers for Disease Control and Prevention (CDC), the best way to prevent long COVID is to avoid getting infected [44]. However, this is not feasible given the high dissemination of coronavirus and its variants. In terms of other methods, vaccination has been found to play an important role in the prevention of long COVID symptoms, with meta-analyses showing that taking at least one dose of a COVID-19 vaccine was associated with a 46–50% decreased risk of long COVID, with two doses possibly being more effective than one dose [45–47]. Similarly, meta-analyses have suggested that people who had vaccines before SARS-CoV-2 infection [46–48] and vaccines after infection [47] were less likely to report long COVID symptoms than unvaccinated people. Similarly, receiving a vaccine after the development of long COVID may also have some effects [46,48]. Therefore, vaccination before and after the SARS-CoV-2 infection, and possibly after the appearance of long COVID, could help with the prevention of long COVID.

### Implications

Our findings have several implications. First, awareness of long COVID-19 should be urgently raised in general, as it is possible that COVID-19 may not be exactly like other transient infections without obvious post-infection symptoms, but could rather have diverse and long-lasting symptoms. Second, SARS-CoV-2 variants still need to be monitored, and the mechanisms by which the long-term symptoms develop and maintain should be further explored and tested empirically. Future longitudinal studies with longer-term follow-up for at least 12 months should be conducted, as should research identifying high-risk groups for having long COVID-19, especially among people with multiple infections or other comorbidities. Third, there is an urgent need for the development and testing of potential interventions and treatment regimens targeting long COVID symptoms. Fourth, health care services should be readied and prepared on time by ensuring the availability of long COVID screening, monitoring, treatment, and rehabilitation. Professional training regarding long COVID symptoms should be provided to health care providers, with a focus on interdisciplinary collaboration due to the manifestation of long COVID symptoms in multiple body systems and organs. Lastly, prevention of infection and continuous vaccination might still be needed to prevent infection and severe outcomes after infection [49].

### Strengths and limitations

Our systematic review and meta-analysis included a relatively large number of studies that measured long COVID-19 symptoms with a control group to assess the excess risk between COVID-19 infection and various long COVID symptoms. We also conducted subgroup analyses to explore the trends in long COVID risk over time, as well as other factors such as age, gender, risk of bias, and adjustment, lending to the robustness of our findings.

However, our study also has limitations. First, there was high heterogeneity in the meta-analysis, which might have come from the lack of a unified consensus on the definition of long COVID, variability in symptom assessment methods, and differences in study populations and settings. However, we observed that most of the studies were on the same side of the forest plots, indicating that COVID-19 infection does increase the risk of certain long COVID symptoms. Second, the risks in our meta-analysis might be underestimated, since some untested participants in the non-COVID-19 control group might have been infected but have had no positive record. Third, while we excluded studies with less than 100 COVID-19 participants to avoid small study effects, this ensured the generalisability of our findings for broader population of COVID-19 patients [50]. Fourth, the power of our meta-analysis in detecting some differences might have been limited, since only a few studies reported on some of the symptoms or measured the difference between genders. Fifth, we did not conduct subgroup analyses for factors such as pre-existing health conditions, severity of initial COVID-19 infection, and different COVID-19 variant waves due to having insufficient data. Finally, long COVID symptoms were rarely measured in specific ethnic groups (e.g. Asian people), possibly because we only looked at English-language studies. Differences in long COVID symptoms between different ethnic groups and their mechanism are worth exploring in the future.

## CONCLUSIONS

In this systematic review and meta-analysis, we found that COVID-19 can significantly increase the risk of various long-term symptoms at least three months after infection. The risk of many long COVID symptoms in the included studies did not vary according to gender, age, or decrease over time beyond three months after the initial infection. Countries and health care systems should provide services and interventions specifically tailored to address the symptoms of long COVID.

## Additional material


Online Supplementary Document
