# Supplementary material for: Excess risks of long COVID symptoms compared with identical symptoms in the general population: A systematic review and meta-analysis of studies with control groups
Source: J Glob Health. 2024 Aug 12;14:05022. doi: 10.7189/jogh.14.05022 (PMC11317913; doi:10.7189/jogh.14.05022)
Supplement: Online Supplementary Document [file jogh-14-05022-s001.pdf]

Material S1. Search strategies

Ovid MEDLINE(R) and Epub Ahead of Print, In-Process, In-Data-Review & Other Non-Indexed Citations 1946 to February 10, 2023

Step	Search term	No. of results
#1	(long covid* or long-term covid*).ti,ab.	2181
#2	post-covid syndrome.ti,ab.	264
#3	exp coronavirus/ or exp coronavirus infections/	229253
#4	(coronavirus or corona virus or coronavirinae).ti,ab.	112470
#5	(covid* or 2019-nCoV or SARS-CoV*).ti,ab.	316039
#6	or/3-5	349758
#7	((post* or persist* or chronic or sustain* or long-term or long-lasting) adj3 (symptom* or condition* or illness or unwell or rehabilitation or fatigue or weakness or cough or sore throat or dyspnea or breathlessness or arthromyalgia or cogniti* or depressi* or anxiety or memory or concentration difficult* or insomnia or sleep or diarrhea or dizziness or anosmia or smell blindness or olfactory impairment or olfactory dysfunction or loss of smell or smell disorder or ageusia or taste blindness or gustatory impairment or gustatory dysfunction or loss of taste or taste disorder or hear loss or alopecia or palpitations or headache or backache or waist pain or joint pain or muscle pain or myalgia)).ti,ab.	281666
#8	6 and 7	7618
#9	1 or 2 or 8	9112
#10	(control* or case-control or compar* or match*).ti,ab.	9314414
#11	(healthy or non-COVID-19 or non-infect* or influenza or respiratory tract infection* or pneumonia or negative).ti,ab.	2433959
#12	("not" adj2 (covid* or 2019-nCoV or SARS-CoV*)).ti,ab.	1361
#13	("without" adj1 (covid* or 2019-nCoV or SARS-CoV*)).ti,ab.	1227
#14	or/10-13	10563571
#15	9 and 14	4097
#16	limit 15 to yr="2020-Current"	4052
#17	exp animals/ not humans/	5088227
#18	16 not 17	4045
#19	limit 18 to english	3977
#20	limit 19 to (review or case reports or comment or clinical trial protocol or meta-analysis or systematic review or editorial)	765
#21	19 not 20	3213

Embase 1910 to Present

Step	Search term	No. of results
#1	(long covid* or long-term covid*).ti,ab.	2642
#2	post-covid syndrome.ti,ab.	398
#3	exp coronavirus/ or exp coronavirus infections/	365593
#4	(coronavirus or corona virus or coronavirinae).ti,ab.	122223
#5	(covid* or 2019-nCoV or SARS-CoV*).ti,ab.	370795
#6	or/3-5	429732
#7	((post* or persist* or chronic or sustain* or long-term or long-lasting) adj3 (symptom* or condition* or illness or unwell or rehabilitation or fatigue or weakness or cough or sore throat or dyspnea or breathlessness or arthromyalgia or cogniti* or depressi* or anxiety or memory or concentration difficult* or insomnia or sleep or diarrhea or dizziness or anosmia or smell blindness or olfactory impairment or olfactory dysfunction or loss of smell or smell disorder or ageusia or taste blindness or gustatory impairment or gustatory dysfunction or loss of taste or taste disorder or hear loss or alopecia or palpitations or headache or backache or waist pain or joint pain or muscle pain or myalgia)).ti,ab.	400064
#8	6 and 7	10219
#9	1 or 2 or 8	12032
#10	(control* or case-control or compar* or match*).ti,ab.	12252926
#11	(healthy or non-COVID-19 or non-infect* or influenza or respiratory tract infection* or pneumonia or negative).ti,ab.	3388293
#12	("not" adj2 (covid* or 2019-nCoV or SARS-CoV*)).ti,ab.	1858
#13	("without" adj1 (covid* or 2019-nCoV or SARS-CoV*)).ti,ab.	1831
#14	or/10-13	13888796
#15	9 and 14	5932
#16	limit 15 to yr="2020-Current"	5845
#17	exp animals/ not humans/	11605138
#18	16 not 17	5832
#19	limit 18 to english	5757
#20	limit 19 to (conference abstracts or book or book series or chapter or meta analysis or systematic review or review or conference review)	2389
#21	19 not 20	3368

CINAHL (EBSCOhost)

Step	Search term	No. of results
S1	AB ("long covid*" OR "long-term covid*" OR "post-covid syndrome")<br>Expanders - Apply equivalent subjects<br>Search modes - Find all my search terms	462
S2	TI ("long covid*" OR "long-term covid*" OR "post-covid syndrome")<br>Expanders - Apply equivalent subjects<br>Search modes - Find all my search terms	578
S3	MH "Coronavirus+" OR MH "Coronavirus Infections+"<br>Expanders - Apply equivalent subjects<br>Search modes - Find all my search terms	44,202
S4	AB (coronavirus OR "corona virus" OR coronavirinae)<br>Expanders - Apply equivalent subjects<br>Search modes - Find all my search terms	24,686
S5	TI (coronavirus OR "corona virus" OR coronavirinae)<br>Expanders - Apply equivalent subjects<br>Search modes - Find all my search terms	8,932
S6	AB (covid* OR "2019-nCoV" OR "SARS-CoV*")<br>Expanders - Apply equivalent subjects<br>Search modes - Find all my search terms	68,341
S7	TI (covid* OR "2019-nCoV" OR "SARS-CoV*")<br>Expanders - Apply equivalent subjects<br>Search modes - Find all my search terms	82,657
S8	S3 OR S4 OR S5 OR S6 OR S7<br>Expanders - Apply equivalent subjects<br>Search modes - Find all my search terms	120,255
S9	AB(post* or persist* or chronic or sustain* or "long-term" or "long-lasting") N3 (symptom* or condition* or illness or unwell or rehabilitation or fatigue or weakness or cough or "sore throat" or dyspnea or breathlessness or arthromyalgia or cogniti* or depressi* or anxiety or memory or "concentration difficult*" or insomnia or sleep or diarrhea or dizziness or anosmia or "smell blindness" or "olfactory impairment" or "olfactory dysfunction" or "loss of smell" or "smell disorder" or ageusia or "taste blindness" or "gustatory impairment" or "gustatory dysfunction" or "loss of taste" or "taste disorder" or "hear loss" or alopecia or palpitations or headache or backache or "waist pain" or "joint pain" or "muscle pain" or myalgia)<br>Expanders - Apply equivalent subjects<br>Search modes - Find all my search terms	106,755

S10	TI (post* or persist* or chronic or sustain* or "long-term" or "long-lasting") N3 (symptom* or condition* or illness or unwell or rehabilitation or fatigue or weakness or cough or "sore throat" or dyspnea or breathlessness or arthromyalgia or cogniti* or depressi* or anxiety or memory or "concentration difficult*" or insomnia or sleep or diarrhea or dizziness or anosmia or "smell blindness" or "olfactory impairment" or "olfactory dysfunction" or "loss of smell" or "smell disorder" or ageusia or "taste blindness" or "gustatory impairment" or "gustatory dysfunction" or "loss of taste" or "taste disorder" or "hear loss" or alopecia or palpitations or headache or backache or "waist pain" or "joint pain" or "muscle pain" or myalgia)<br>Expanders - Apply equivalent subjects<br>Search modes - Find all my search terms	35,683
S11	S9 OR S10<br>Expanders - Apply equivalent subjects<br>Search modes - Find all my search terms	124,487
S12	S8 AND S11<br>Expanders - Apply equivalent subjects<br>Search modes - Find all my search terms	2,701
S13	S1 OR S2 OR S12<br>Expanders - Apply equivalent subjects<br>Search modes - Find all my search terms	3,271
S14	AB (control* OR "case-control" OR compar* OR match*)<br>Expanders - Apply equivalent subjects<br>Search modes - Find all my search terms	1,532,137
S15	TI (control* OR "case-control" OR compar* OR match*)<br>Expanders - Apply equivalent subjects<br>Search modes - Find all my search terms	361,239
S16	AB (healthy OR "non-COVID-19" OR "non-infect*" OR influenza OR "respiratory tract infection*" OR pneumonia OR negative)<br>Expanders - Apply equivalent subjects<br>Search modes - Find all my search terms	461,740
S17	TI (healthy OR "non-COVID-19" OR "non-infect*" OR influenza OR "respiratory tract infection*" OR pneumonia OR negative)<br>Expanders - Apply equivalent subjects<br>Search modes - Find all my search terms	103,980
S18	AB ("not" N2 (covid* OR "2019-nCoV" OR "SARS-CoV*"))<br>Expanders - Apply equivalent subjects<br>Search modes - Find all my search terms	68,341
S19	TI ("not" N2 (covid* OR "2019-nCoV" OR "SARS-CoV*"))<br>Expanders - Apply equivalent subjects<br>Search modes - Find all my search terms	82,657
S20	AB ("without" N1 (covid* OR "2019-nCoV" OR "SARS-CoV*"))<br>Expanders - Apply equivalent subjects<br>Search modes - Find all my search terms	550

S21	TI ("without" N1 (covid* OR "2019-nCoV" OR "SARS-CoV*"))<br>Expanders - Apply equivalent subjects<br>Search modes - Find all my search terms	116
S22	S14 OR S15 OR S16 OR S17 OR S18 OR S19 OR S20 OR S21<br>Expanders - Apply equivalent subjects<br>Search modes - Find all my search terms	2,026,916
S23	S13 AND S22<br>Expanders - Apply equivalent subjects<br>Search modes - Find all my search terms	3,173
S24	PT (book OR book chapter OR case study OR meta analysis OR protocol OR randomized controlled trial OR review OR systematic review)<br>Expanders - Apply equivalent subjects<br>Search modes - Find all my search terms	1,157,109
S25	S23 NOT S24<br>Expanders - Apply equivalent subjects<br>Search modes - Find all my search terms<br>Limiters - Published Date: 20200101-; English Language	2727

ProQuest Coronavirus Research Database

Step	Search term	No. of results
#1	summary((("long covid*" OR "long-term covid*" OR "post-covid syndrome") OR ((coronavirus OR "corona virus" OR coronavirinae OR covid* OR 2019-nCoV OR SARS-CoV*) AND ((post* OR persist* OR chronic OR sustain* OR long-term OR long-lasting) PRE/3 (symptom* OR condition* OR illness OR unwell OR rehabilitation OR fatigue OR weakness OR cough OR "sore throat" OR dyspnea OR breathlessness OR arthromyalgia OR cogniti* OR depressi* OR anxiety OR memory OR "concentration difficult*" OR insomnia OR sleep OR diarrhea OR dizziness OR anosmia OR "smell blindness" OR "olfactory impairment" OR "olfactory dysfunction" OR "loss of smell" OR "smell disorder" OR ageusia OR "taste blindness" OR "gustatory impairment" OR "gustatory dysfunction" OR "loss of taste" OR "taste disorder" OR "hear loss" OR alopecia OR palpitations OR headache OR backache OR "waist pain" OR "joint pain" OR "muscle pain" OR myalgia)))) AND (control* OR case-control OR compar* OR match* OR healthy OR non-COVID-19 OR non-infect* OR influenza OR "respiratory tract infection*" OR pneumonia OR negative))<br><br>Additional limits:<br>Date: After 01 January 2020; Language: English	1314

WHO COVID-19 Research Database

Step	Search term	No. of results
#1	(("long covid*" OR "long-term covid*" OR "post-covid syndrome") OR ((coronavirus OR "corona virus" OR coronavirinae OR covid* OR "2019-nCoV" OR "SARS-CoV*") AND ((post* OR persist* OR chronic OR sustain* OR "long-term" OR "long-lasting") PRE/3 (symptom* OR condition* OR illness OR unwell OR rehabilitation OR fatigue OR weakness OR cough OR "sore throat" OR dyspnea OR breathlessness OR arthromyalgia OR cogniti* OR depressi* OR anxiety OR memory OR "concentration difficult*" OR insomnia OR sleep OR diarrhea OR dizziness OR anosmia OR "smell blindness" OR "olfactory impairment" OR "olfactory dysfunction" OR "loss of smell" OR "smell disorder" OR ageusia OR "taste blindness" OR "gustatory impairment" OR "gustatory dysfunction" OR "loss of taste" OR "taste disorder" OR "hear loss" OR alopecia OR palpitations OR headache OR backache OR "waist pain" OR "joint pain" OR "muscle pain" OR myalgia)))) AND (control* OR "case-control" OR compar* OR match* OR healthy OR "non-COVID-19" OR "non-infect*" OR influenza OR "respiratory tract infection*" OR pneumonia OR negative)<br><br>Additional limits:<br>In Title, abstract, and subject<br>Language-English<br>Topics- Long Covid<br>Document type- Article type & Preprint	2347

Material S2. Classification of long COVID symptoms

Category	Symptom	Names of symptoms used in different studies
General	Post-exertional malaise	Post-exertional malaise
	Fatigue	Fatigue; fatigue or weakness; fatigue or asthenia; fatigue or exhaustion; fatigue, tiredness, or exhaustion; chronic fatigue; chronic fatigue syndrome; tiredness; general tiredness; extreme tiredness-lack of energy; screen fatigue; weakness or tiredness
	Swelling of legs	Limb swelling
	Fever, sweats, or chills	Fever; Feverishness; Chills and fever; fever or feverish
Neurologic	Smell	Loss of smell; Change in smell; Loss or change in smell; Loss of sense of smell; Smell disorder; ansomia; Hyposmia; Dysosmia
	Taste	Loss of taste; Change in taste; Loss or change in taste; Loss of taste; Loss of sense of taste; Taste disorder; Ageusia; Hypogeusia; Dysgeusia
	Brain fog	Brain fog
	Cognitive decline	cognitive decline; cognition; Cognitive complaints; Cognitive symptoms; Dementia; Vascular dementia; Memory problems; Poor memory; Memory impairment; Impaired memory; Disturbance of memory; Confusion memory loss; Problems with memory; Problems with long-term memory; Trouble remembering or concentrating; Concentration and memory; Difficulty concentrating or loss of memory; Forgetfulness or memory problems; Forgetfulness; Amnesia
	Dizziness	Dizziness; Vertigo or dizziness; Vertigo; Peripheral or central vertigo; Dizziness or lightheaded; Dizziness or lack of equilibrium; Dizziness or light-headedness; Presyncope or dizziness; Dizziness or fainting
	Headache	Headache
	Neurological problems	Neurologic; Neurological disorders; Neuralgia
Respiratory	Shortness of breath	Shortness of breath; Shortness of breath - resting; Dyspnea; Dyspnea or shortness of breath; Difficulties with breathing; Difficulty breathing, short of breath; Breathlessness; Breathless; Trouble breathing; Abnormal breathing
	Cough	Cough, Coughing, Dry cough, Persistent cough
	Throat pain	Sore throat; Sore throat or difficult to swallow
Cardiac	Chest pain	Chest pain; Nonspecific chest pain; Unusual chest pain; Chest pain or chest tightness; Thoracic pain
	Palpitations	Palpitation; Heart palpitation; Palpitations or tachycardia
Dermatologic	Hair loss	Hair loss; Loss of hair; Alopecia
	Skin rash	Skin rash; Rash; Purpura or rash; Rash and other nonspecific skin eruption; Skin condition or rash
Eye	Vision	Visual disturbances; Blurred vision
	Itchy eyes	Itchy eyes
Ear	Hearing	Hearing problems; Problems hearing; Hearing loss; Sensorineural hearing loss

Musculoskeletal	Muscle pain	Muscle pain; muscle aches; Muscle aches or muscle pains; Painful muscles; Myalgia; Muscle aches/weakness; Unusual strong muscle pains; Fibromyalgia
	Joint pain	Joint pain; Arthralgia
	Back pain	Back pain
Psychiatric	Sleep problems	Sleep; Sleep disturbances; Sleep problems; Sleep-wake disorders; Sleep disorder; Sleep difficulties; Sleeping disorder; Sleep quality; Insomnia
	Depression	Depression; Depressive symptoms; Depression(abnormal); Depressive disorders; Major depressive disorder
	Anxiety	Anxiety; Anxiety symptoms; Anxiety(abnormal); Anxiety disorder; Mood/anxiety; Low mood/anxiety; Anxiety and fear-related disorders
Gastrointestinal	Gastrointestinal symptoms	Gastrointestinal; Gastrointestinal disturbances; Diarrhoea; Diarrhoea or constipation
	Abdominal pain	Abdominal pain; Unusual abdominal pain; tummy ache

Material S3. Newcastle-Ottawa Scale, adapted for quality assessment of the long COVID systematic review

Selection

1) Representativeness of the COVID-19 participants (sample)
   a) truly or somewhat representative of the COVID-19 patients in the community ✷
   b) truly or somewhat representative of the COVID-19 patients in the hospital ✷
   c) selected group of users e.g. nurses, volunteers
   d) no description of the derivation of the COVID-19 patients

2) Selection of the non-COVID-19 participants (control)
   a) drawn from the same community/hospital as the COVID-19 participants ✷
   b) drawn from a different source
   c) no description of the derivation of the non-COVID-19 participants

3) Ascertainment of COVID-19 infection
   a) laboratory-confirmed, clinically diagnosed ✷
   b) medical records ✷
   c) self-report, e.g. rapid antigen test
   d) no description

4) Definition of non-COVID-19 participants
   a) laboratory-confirmed ✷
   b) medical records ✷
   c) self-report, e.g. rapid antigen test
   d) no description

Comparability

5) Comparability of COVID-19 and non-COVID-19 participants on the basis of the design or analysis
   a) study controls for the most important factors, e.g. age and gender ✷
   b) study controls for any additional factor, e.g. chronic condition(s) ✷

Outcome

6) Assessment of long COVID symptoms
   a) clinically diagnosed ✷
   b) medical records ✷
   c) validated scales ✷
   d) self-reported ✷
   e) no description

7) Same method of ascertainment for COVID-19 positive and negative participants
   a) yes ✷
   b) no

8) Non-response rate/rate of loss to follow-up: __________

a) for case-control and cross-sectional studies, comparability between respondent and non-respondent characteristics is established, or the response rate is >70% ✷

b) for case-control and cross-sectional studies, the comparability between respondents and non-respondents is unsatisfactory, and the response rate is <70%

c) for cohort studies, subjects lost to follow up unlikely to introduce bias e.g. small number lost (>70% follow-up), or description provided of those lost ✷

d) for cohort studies, follow up rate < 70% and no description of those lost

e) no statement

Table S1. Characteristics of included studies

	Study	Design	Country	Sources of participants	COVID-19 participants				Control			Follow-up time	Follow-up mode	Symptoms
					n	COVID-19 Confirmation	Mean Age	Female (%)	n	Mean Age	Female (n,%)			
1	Chevinsky (2021)[1]	Retrospective cohort	USA	Premier Healthcare Database Special COVID-19 Release data	27284	Medical record	NA	52.5	27284	NA	52.6	90-120 days	Medical record	Chest pain, anxiety, depression, sleep problems
2	Estiri (2021)[2]	Retrospective cohort	USA	Electronic health record diagnosis records	22475	PCR	48	63.9	73550	51.5	63.9	3-6 and 6-9 months	Medical record	Fatigue, chest pain, palpitations, hair loss, skin rash, hearing, vision, dizziness, depression, shortness of breath or difficulties with breathing, neurological problem, cognitive decline
3	Havervall (2021)[3]	Prospective cohort	Sweden	Health care professionals at a hospital	323	Seropositive for SARS-CoV-2 anti-spike IgG	43[a]	83.0	1072	47[a]	96.0	≥4 months, ≥8 months	Online survey with smart phone app	Fatigue, palpitations, headache, smell, taste, shortness of breath or difficulties with breathing, cognitive decline, at least one symptom, sleep problems
4	Liu (2021)[4]	Cross-sectional study	China	Inpatients discharged from COVID-19-designated hospitals	1539	World Health Organization interim guidance	69[a]	52.0	466	67[a]	51.5	6 months	Telephone interview	Cognitive decline
5	Riestra-Ayora (2021)[5]	Prospective cohort	Spain	Health workers from a tertiary care hospital	195	RT-PCR	41.62	80.0	125	46.5	84.8	6 months	NA	Muscle pain, headache, smell, taste, cough, shortness of breath or difficulties with breathing
6	Soraas (2021)[6]	Prospective cohort	Norway	Adults tested for SARS-CoV-2 in four large accredited laboratories	794	RT-PCR	47.3	54.0	7992	44.8	75.0	3-8 months	Online follow-up questionnaire	Fatigue, fever, sweats, or chills, headache, cough, shortness of breath or difficulties with breathing, throat pain, at least one symptom
7	Taquet (2021)[7]	Retrospective cohort	UK	Electronic health records data	106578	Electronic health records	39.4	58.4	106578	38.3	57.6	3-6 months	Electronic health records	Fatigue, muscle pain, headache, shortness of breath or difficulties with breathing, cognitive decline, at least one symptom
8	Al-Aly (2022)[8]	Retrospective cohort	USA	US Veterans Health Administration electronic health databases	33940	Health record	62.82	11.15	4983491	62.77	10.95	90-180 days	Health record	Fatigue, neurological problem, at least one symptom
9	Alessia (2022)[9]	Prospective cohort	Switzerland	Primary and secondary school children	230	Serology testing	Range: 8-16	NA	331	Range: 8-16	NA	6-9 months	Online questionnaires	At least one symptom
10	Ali (2022)[10]	Retrospective cohort	Bangladesh	Participants lived at the community level	439	Laboratory-tested COVID-19	38.3	49.2	439	38.3	51.7	91-180 days, >180 days	Interviewed face to face using a paper-based semi-structured questionnaire	Headache
11	Ballering (2022)[11]	Prospective cohort	Netherlands	People in the north of the Netherlands	4231	PCR or physician's diagnosis	52.4	65.7	8462	54.0	65.7	90–150 days	Online survey	Fatigue, fever, sweats, or chills, chest pain, gastrointestinal symptoms, back pain, muscle pain, dizziness, headache, cough, shortness of breath or difficulties with breathing, throat pain, itchy eyes, at least one symptom
12	Ballouz (2022)[12]	Prospective cohort	Switzerland	General population	1543	Laboratory-confirmed	49.0[a]	50.6	628	65.0[a]	51.3	6 months, 12 months	Questionnaires	Fatigue, fever, sweats, or chills, post-exertional malaise, chest pain, palpitations, hair loss, skin rash, hearing, vision, gastrointestinal symptoms, joint pain, muscle pain, dizziness, headache, anxiety, depression, cough, shortness of breath or difficulties with breathing, cognitive decline, sleep problems
13	Berg (2022)[13]	Cross-sectional study	Denmark	Danish adolescents aged 15–18 years	6630	Danish COVID-19 database	17.6[a]	58.4	21640	17.5[a]	57·3	3, 6, 9, 12 months	Online survey	Fatigue, fever, sweats, or chills, chest pain, palpitations, skin rash, dizziness, headache, cough, shortness of breath or difficulties with breathing, throat pain, cognitive decline
14	Berg (2022)[14]	Cross-sectional study	Denmark	Danish children aged 0-14 years	10997	PCR	10.2[a]	48.2	33016	10.6[a]	48.3	3, 6, 9, 12 months	Online survey	Fatigue, fever, sweats, or chills, chest pain, palpitations, dizziness, headache, cough, shortness of breath or difficulties with breathing, throat pain, cognitive decline
15	Bergia (2022)[15]	Retrospective cohort	Spain	Children under 18 years old at university hospitals	451	PCR, antigen test, serology	5.9	45	98	7.8	43	351 days (IQR 330-471 days)	Telephone questionnaire	At least one symptom
16	Bertran (2022)[16]	Prospective cohort	UK	Adolescents aged 11–17 years from the Public Health England database	6334	PCR	Range:11–17	NA	6454	NA	NA	6 months	Questionnaire	At least one symptom
17	Bsteh (2022)[17]	Prospective cohort	Austria	Austrian MS-COVID-19 registry	211	PCR	42.6	69.2	211	43.4	69.2	3,6,12 months	Medical records	Fatigue, smell, shortness of breath or difficulties with breathing
18	Buonsenso (2022)[18]	Prospective cohort	Italy	Children and their household contacts	Children: 138<br>Adults: 107	PCR	Children: 10.6<br>Adults: 44.6	Children: 61.5<br>Adults: 45.7	Children: 37<br>Adults: 49	Children: 10.5<br>Adults: 42.3	Children: 52.6<br>Adults: 46.3	6-9 months	Telephone and face-to-face visits	Gastrointestinal symptoms, at least one symptom, sleep problems

	Study	Design	Country	Sources of participants	COVID-19 participants				Control			Follow-up time	Follow-up mode	Symptoms
					n	COVID-19 Confirmation	Mean Age	Female (%)	n	Mean Age	Female (n,%)			
19	Caspersen (2022)[19]	Retrospective cohort	Norway	General population	774	PCR	NA	58	72953	NA	59.3	11-12 months	Electronic questionnaire	Fatigue, fever, sweats, or chills, chest pain, palpitations, hair loss, skin rash, joint pain, muscle pain, brain fog, dizziness, headache, anxiety, depression, cough, shortness of breath or difficulties with breathing, cognitive decline, sleep problems
20	Castro (2022)[20]	Retrospective cohort	USA	Individuals discharged from 6 hospitals	5771	PCR	NA	NA	30193	NA	NA	90-150 days	Medical record	Fatigue, headache, smell, anxiety, cognitive decline, sleep problems
21	Clift (2022)[21]	Retrospective cohort	UK	Adults identified from QResearch primary care database	32525	Electronic health record databases	65.4	46.48	8330986	49.07	49.86	12 months	Electronic health record databases	Anxiety, depression, cognitive decline
22	Desgranges (2022)[22]	Prospective cohort	Switzerland	Outpatients during the initial visit in the emergency department (ED), in the SARS-CoV-2 screening center and in two outpatient clinics	418	PCR	41[a]	62.4	89	36[a]	64.0	3-10 months	Phone interview	Fatigue, chest pain, hair loss, vision, sweats, or chills, headache, cough, shortness of breath or difficulties with breathing, cognitive decline, at least one symptom, sleep problems
23	Fjelltveit (2022)[23]	Prospective cohort	Norway	Home-isolated patients tested at the city's centralized testing facility	233	RT-PCR	44[a]	53.2	189	41[a]	65.6	6, 12, 18 months	Clinical follow-up (questionnaire)	Fatigue, shortness of breath or difficulties with breathing, cognitive decline
24	Funk (2022)[24]	Prospective cohort	8 countries	36 emergency departments in 8 countries	1686	Nucleic acid testing	3[a]	47.2	1321	NA	52.9	90-120 days	Telephone or email or text surveys	Fatigue, fever, sweats, or chills, chest pain, skin rash, gastrointestinal symptoms, dizziness, headache, anxiety, depression, cough, shortness of breath or difficulties with breathing, neurological problem, at least one symptom
25	Haddad (2022)[25]	Prospective cohort	Germany	Households	Adults: 494; Adolescents 14-18: 59; Children<14: 151	RT-PCR or antibody tests	Adults: 45[a]; Adolescents 14-18: 16[a]; Children<14: 8[a]	adults: 52.2; Adolescents 14-18: 47.5; children<14: 46.4	adults: 229; Adolescents 14-18: 81; children<14: 253	adults: 43[a]; Adolescents 14-18: 16[a]; children<14: 7[a]	adults: 49.3; Adolescents 14-18: 55.6); children<14: 50.6	11-12 months	Online questionnaires	Fatigue, hair loss, anxiety, shortness of breath or difficulties with breathing, cognitive decline, at least one symptom, sleep problems
26	Hastie (2022)[26]	Prospective cohort	UK	Every adult (>16 years) in Scotland	Symptomatic: 31486 asymptomatic: 1795	PCR	Asymptomatic 43[a] Symptomatic 44[a]	Asymptomatic 53.31, Symptomatic 64.16	62957	45[a]	59.44	6-18 months	Online survey	Fatigue, chest pain, palpitations, skin rash, hearing, abdominal pain, gastrointestinal symptoms, joint pain, muscle pain, headache, smell, taste, cough, shortness of breath or difficulties with breathing, at least one symptom, sleep problems
27	Huang (2022)[27]	Prospective cohort	China	Individuals discharged from hospital	1127	Laboratory confirmed COVID-19	59.0[a]	46.1	1127	59.0[a]	46.1	2 years	Telephone survey	Chest pain, palpitations, hair loss, skin rash, joint pain, muscle pain, dizziness, headache, smell, taste, cough, shortness of breath or difficulties with breathing, throat pain, at least one symptom, sleep problems
28	Liptak (2022)[28]	Prospective cohort	Slovakia	Adult patients from a outpatient COVID-19 testing center	205	PCR	NA	60.5	132	41[a]	65.2	5–8 months	Telephone survey	Abdominal pain, gastrointestinal symptoms
29	Magnusdottir (2022)[29]	Prospective cohort	six countries (Denmark, Estonia, Iceland, Norway, Sweden, and the UK)	Individuals aged 18 years and older	9979	Self-reports of a confirmed positive RT-PCR test or positive antibody test	46.6	67.9	237270	48.9	61.7	6-16 months	Validated mental health instruments	Anxiety, depression, sleep problems
30	Magnusson (2022)[30]	Prospective cohort	Norway	All persons aged 18-70 years living in Norway	36396	PCR	39.0	49.5	105196	42.0	50.3	90-126 days	Medical record	Fatigue, palpitations, brain fog, cough, shortness of breath or difficulties with breathing
31	Marasco (2022)[31]	Prospective cohort	14 countries: Italy, Bangladesh, Cyprus, Egypt, Israel, India, Macedonia, Malaysia, Romania, the Russian Federation, Serbia, Spain, Sweden and Turkey	Hospitalised patients	614	Laboratory-confirmed SARS-CoV-2 infection	49.9	40.7	269	50.9	39.0	6 and 12 months	Questionnaire/ e-Case report	Abdominal pain, gastrointestinal symptoms, anxiety, depression
32	Nehme (2022)[32]	Prospective cohort	Switzerland	Outpatient symptomatic individuals tested for SARS-CoV-2 at the Geneva University Hospitals	287	RT-PCR	44.2	64.8	1160	45.5	60.3	12 months	Online questionnaire	Fatigue, chest pain, palpitations, joint pain, muscle pain, dizziness, headache, smell, taste, cough, shortness of breath or difficulties with breathing, cognitive decline, at least one symptom, sleep problems

	Study	Design	Country	Sources of participants	COVID-19 participants				Control			Follow-up time	Follow-up mode	Symptoms
					n	COVID-19 Confirmation	Mean Age	Female (%)	n	Mean Age	Female (n,%)			
33	Noviello (2022)[33]	Prospective cohort	Italy	Patients, employees and healthcare professionals aged 18-60 years	164	PCR	44.1	40.2	183	39.6	60.7	4.8 ± 0.3 months	Online structured questionnaire	Fatigue, back pain, headache, anxiety, depression, sleep problems
34	Nugawela (2022)[34] Stephenson (2022)[35] Pereira (2023)[36]	Prospective cohort	UK	Children and young people aged 11–17 years in Public Health England database	6407	PCR	Range: 11-17	62.3	6542	Range: 11-17	62.9	3, 6 months	Questionnaire	Fatigue, fever, sweats, or chills, chest pain, abdominal pain, gastrointestinal symptoms, muscle pain, dizziness, headache, smell, cough, shortness of breath or difficulties with breathing, throat pain, at least one symptom,
35	Rivera-Izquierdo (2022)[37]	Prospective cohort	Spain	Hospitalised patients in 4 hospitals and 29 primary care centres in Andalusia	453	Laboratory-confirmed SARS-CoV-2 infection through nasopharyngeal PCR	61.2	42.6	453	55.9	53.2	12 months	Telephone survey	Fatigue, chest pain, abdominal pain, gastrointestinal symptoms, headache, anxiety, depression, shortness of breath or difficulties with breathing, cognitive decline, at least one symptom, sleep problems
36	Robineau (2022)[38]	Prospective cohort	France	French adult population affiliated with the National Fund for Health Insurance	1022 (ECDC+/Sero + 494, ECDC-/Sero+ 528)	Dried blood spot (DBS) test for anti-SARS-CoV-2 antibodies	ECDC+/Sero + 42.0[a] ECDC-/Sero+ 39.5[a]	59.1	24888 (ECDC+/Sero - 3534, ECDC-/Sero- 21354)	ECDC+/Sero- 47.0[a] ECDC-/Sero- 51.5[a]	51.0	242 days [IQR:238−251]	Online survey	Fever, sweats, or chills, chest pain, palpitations, abdominal pain, gastrointestinal symptoms, back pain, joint pain, muscle pain, dizziness, headache, cough, shortness of breath or difficulties with breathing, cognitive decline, at least one symptom, sleep problems
37	Sandmann (2022)[39]	Prospective cohort	UK	People aged 12–85 years from the Second Generation Surveillance System	548	PCR	41.1	61.5	651	45.4	70.7	6 months	Survey	Fatigue, fever, sweats, or chills, skin rash, abdominal pain, gastrointestinal symptoms, muscle pain, headache, cough, shortness of breath or difficulties with breathing, throat pain, at least one symptom
38	Sorensen (2022)[40] Spiliopoulos (2022)[41]	Cross-sectional study (Sorensen) Prospective cohort (Spiliopoulos)	Denmark	All residents above 15 years of age	61002	RT-PCR	49[a]	58.7	91878	53[a]	62.8	4, 6, 9, 12 months	Web-based questionnaires distributed via the national "e-Boks" system	Fatigue, fever, sweats, or chills, chest pain, abdominal pain, gastrointestinal symptoms, muscle pain, sweats, or chills, dizziness, headache, smell, taste, anxiety, depression, cough, shortness of breath or difficulties with breathing, throat pain, cognitive decline, at least one symptom, sleep problems
39	Spatz (2022)[42] Wisk (2022)[43]	Prospective cohort	USA	Participants who underwent testing for SARS-CoV-2 infection to diagnose acute symptoms	1006	Molecular or antigen-based assay approved or authorized by the US Food and Drug Administration	Overall 41.5	66.0	417	Overall 41.5	66.9	3 months	Electronic surveys sent via text or email	Fatigue, fever, sweats, or chills, chest pain, palpitations, hair loss, abdominal pain, gastrointestinal symptoms, joint pain, muscle pain, dizziness, headache, smell, taste, anxiety, depression, cough, shortness of breath or difficulties with breathing, throat pain, cognitive decline, at least one symptom, sleep problems
40	Strahm (2022)[44]	Prospective cohort	Switzerland	Hospital employees from 23 healthcare institutions	784 (Positive NPS n = 556, Only Seropositive n = 228)	PCR or RAT	Positive NPS 38.9[a], Only Seropositive 37.9[a]	81.0	2550	41.0[a]	78.5	12-24 weeks, >24weeks (median 117 days, IQR 93-147)	Online survey	Fatigue, fever, sweats, or chills, chest pain, palpitations, hair loss, joint pain, dizziness, headache, cough, shortness of breath or difficulties with breathing
41	Subramanian (2022)[45]	Retrospective cohort	UK	A UK-based primary care database	486149	UK-based primary care database	44.1	55.2	1944580	43.8	55.3	>3 months (0.29 years, IQR 0.24–0.42)	UK-based primary care database	Fatigue, fever, sweats, or chills, swelling of legs, chest pain, palpitations, hair loss, skin rash, hearing, abdominal pain, gastrointestinal symptoms, joint pain, muscle pain, brain fog, dizziness, headache, smell, taste, anxiety, depression, cough, shortness of breath or difficulties with breathing, throat pain, cognitive decline, itchy eyes, sleep problems
42	Tartof (2022)[46]	Retrospective cohort	USA	Patients of all ages from 8 large integrated health care systems	127859	Positive result in laboratory testing including RT-PCR and antigen tests	41.2	53.7	127859	41.2	53.7	4-6 months	Electronic medical record	Hair loss, abdominal pain, headache, anxiety, cough, shortness of breath or difficulties with breathing, cognitive decline, sleep problems
43	van der Maaden (2022)[47]	Prospective cohort	Netherlands	Children (ages 5–17) and adults (18 years and above) from community health testing	9116	PCR or antigen SARS-COV-2 test	49.0[a]	63.7	1698	55.3[a]	65.7	3 months	Online survey	Fatigue, fever, sweats, or chills, post-exertional malaise, chest pain, palpitations, skin rash, abdominal pain, brain fog, dizziness, headache, smell, taste, anxiety, depression, cough, shortness of breath or difficulties with breathing, throat pain, neurological problem, cognitive decline, at least one symptom, sleep problems

	Study	Design	Country	Sources of participants	COVID-19 participants				Control			Follow-up time	Follow-up mode	Symptoms
					n	COVID-19 Confirmation	Mean Age	Female (%)	n	Mean Age	Female (n,%)			
44	Bernas (2023)[48]	Cross-sectional study	Germany	Registered potential stem cell donors	11861	PCR	38[a]	74.0	186768	38[a]	69.7	3,6,9,12,15 months	Online survey	Fatigue, chest pain, palpitations, abdominal pain, gastrointestinal symptoms, joint pain, muscle pain, dizziness, headache, anxiety, depression, cough, shortness of breath or difficulties with breathing, throat pain, cognitive decline, sleep problems
45	Merikanto (2023)[49]	Cross-sectional study	16 countries (Austria, Brazil, Bulgaria, Canada, Hong Kong/China, Croatia, Finland, France, Germany, Israel, Italy, Japan, Norway, Portugal, Sweden, USA	General population of adults 18 years old	2705	C-19 antigen/PCR	Asymptomatic 46.6, Mild, 40.7, Moderate 47.8, Severe/ life-threatening 58.1	Asymptomatic 50.1, Mild 55.8, Moderate 56.7, Severe/ life-threatening 42.1%	10923	47.2	52.4	at least 3 months	Online survey	Fatigue, fever, sweats, or chills, post-exertional malaise, palpitations, abdominal pain, gastrointestinal symptoms, brain fog, dizziness, headache, smell, shortness of breath or difficulties with breathing, cognitive decline, sleep problems
46	Miller (2023)[50]	Cross-sectional study	USA	Noninstitutionalized U.S. adults	384	Reported a positive test	Range: 18-70+	55.7	2867	Range: 18-70+	51.5	3-6, 6-9. 9-12, 12+ months	Online survey	At least one symptom
47	Mizrahi (2023)[51]	Retrospective cohort	Israel	Electronic medical records from an Israeli nationwide healthcare organisation	170280	PCR	24[a]	49.6	170280	24[a]	49.6	180-360 days (median 304 days, IQR 128-369 days)	Electronic medical records	Chest pain, palpitations, hair loss, skin rash, vision, abdominal pain, gastrointestinal symptoms, joint pain, muscle pain, dizziness, headache, anxiety, depression, cough, shortness of breath or difficulties with breathing, throat pain, cognitive decline, sleep problems
48	Pihlaja (2023)[52]	Prospective cohort	Finland	Adults aged 18 years or older recruited at an hospital	184	Hospital discharge (ICU and WARD groups) or positive PCR test	53.4	56.0	53	54.9	50.9	3, 6 months	Questionnaire	Depression
49	Seery (2023)[53]	Retrospective cohort	Argentina	Children and adolescents admitted to a hospital	639	PCR	7[a]	46.6	577	8[a]	48.0	6 months	Online survey by parents	Fatigue, palpitations, gastrointestinal symptoms, muscle pain, dizziness, headache, taste, anxiety, depression, cough, shortness of breath or difficulties with breathing, cognitive decline, at least one symptom
50	Shah (2023)[54]	Retrospective cohort	UK	Primary care electronic health record data	11015	Medical record	50.5	61.6	18098	53.6	63.0	Median 136 days (IQR 59, 246)	Electronic health record data	Fatigue, fever, sweats, or chills, swelling of legs, chest pain, palpitations, skin rash, abdominal pain, gastrointestinal symptoms, joint pain, muscle pain, dizziness, headache, cough, shortness of breath or difficulties with breathing, throat pain
51	van Wijhe (2023)[55]	Cross-sectional study	Denmark	Adult Danish general population	742	Previously registered positive PCR tests	48.2	67.0	7420	48.2	67.0	>12 weeks	Web-based questionnaire	Fatigue, gastrointestinal symptoms, muscle pain, dizziness, headache, smell, taste, cough, shortness of breath or difficulties with breathing, cognitive decline

[a] Median

Table S2. Risk of bias of included studies using the adapted Newcastle-Ottawa Scale

	Study	1) Represen-tativeness of the COVID-19 participants (sample)	2) Selection of the non-COVID-19 participants (control)	3) Ascertainment of COVID-19 infection	4) Definition of non-COVID-19 participants	5a) Comparability of COVID-19 and non-COVID-19 participants on the basis of the design or analysis	5b) Comparability of COVID-19 and non-COVID-19 participants on the basis of the design or analysis	6) Assessment of long COVID symptoms	7) Same method of ascertainment for COVID-19 positive and negative participants	8) Non-response rate/rate of loss to follow-up	Total score	Risk of bias
1	Chevinsky (2021)[1]	✷b	✷a	✷b	✷b	✷a	✷b	✷b	✷a	✷c	9	Low
2	Estiri (2021)[2]	✷a	✷a	✷a	✷a	NIL	NIL	✷b	✷a	d	6	Moderate
3	Havervall (2021)[3]	c	✷a	✷a	✷a	NIL	NIL	✷d	✷a	✷c	6	Moderate
4	Liu (2021)[4]	✷b	✷a	✷a	✷a	✷a	✷b	✷c	✷a	✷a	9	Low
5	Riestra-Ayora (2021)[5]	c	✷a	✷a	✷a	✷a	✷b	✷d	✷a	✷a	8	Low
6	Soraas (2021)[6]	c	✷a	✷a	d	NIL	NIL	✷d	✷a	✷c	5	Moderate
7	Taquet (2021)[7]	✷a	✷a	✷b	✷b	✷a	✷b	✷b	✷a	✷c	9	Low
8	Al-Aly (2022)[8]	✷a	✷a	✷b	✷b	✷a	✷b	✷b	✷a	✷c	9	Low
9	Alessia (2022)[9]	c	✷a	✷a	✷a	NIL	NIL	✷d	✷a	✷c	6	Moderate
10	Ali (2022)[10]	c	✷a	✷a	✷a	NIL	NIL	✷c	✷a	✷a	6	Moderate
11	Ballering (2022)[11]	✷a	✷a	✷a	d	✷a	✷b	✷d	✷a	d	7	Low
12	Ballouz (2022)[12]	✷a	✷a	✷a	✷a	✷a	✷b	✷d	✷a	✷c	9	Low
13	Berg (2022)[13]	✷a	✷a	✷a	✷b	✷a	NIL	✷d	✷a	✷a	8	Low
14	Berg (2022)[14]	✷a	✷a	✷a	✷b	✷a	NIL	✷d	✷a	✷a	8	Low
15	Bergia (2022)[15]	c	✷a	✷a	✷b	NIL	NIL	✷d	✷a	✷c	6	Moderate
16	Bertran (2022)[16]	✷a	✷a	✷a	✷a	✷a	✷b	✷c	✷a	✷c	9	Low
17	Bsteh (2022)[17]	c	b	✷a	✷b	✷a	✷b	e	✷a	✷c	6	Moderate
18	Buonsenso (2022)[18]	c	✷a	✷a	✷a	NIL	NIL	✷d	✷a	d	5	Moderate
19	Caspersen (2022)[19]	✷a	✷a	✷a	✷b	✷a	✷b	✷d	✷a	✷c	9	Low
20	Castro (2022)[20]	✷b	✷a	✷a	✷a	✷a	✷b	✷b	✷a	✷a	9	Low
21	Clift (2022)[21]	✷b	✷a	✷a	✷a	✷a	✷b	✷b	✷a	✷c	9	Low
22	Desgranges (2022)[22]	c	✷a	✷a	✷a	✷a	✷b	✷d	✷a	✷c	8	Low
23	Fjelltveit (2022)[23]	c	✷a	✷a	✷a	✷a	✷b	✷c	✷a	✷a	8	Low
24	Funk (2022)[24]	✷a	✷a	✷a	✷a	✷a	✷b	✷d	✷a	✷c	9	Low
25	Haddad (2022)[25]	c	✷a	✷a	✷a	✷a	✷b	✷d	✷a	d	7	Low
26	Hastie (2022)[26]	✷a	✷a	✷a	✷a	✷a	✷b	✷d	✷a	d	8	Low
27	Huang (2022)[27]	✷b	b	✷a	d	✷a	✷b	✷d	✷a	d	6	Moderate
28	Liptak (2022)[28]	c	✷a	✷a	✷a	NIL	NIL	✷c	✷a	d	5	Moderate
29	Magnusdottir (2022)[29]	✷a	✷a	c	d	NIL	NIL	✷c	✷a	✷c	5	Moderate
30	Magnusson (2022)[30]	✷a	✷a	✷a	✷a	✷a	✷b	✷b	✷a	✷c	9	Low
31	Marasco (2022)[31]	c	✷a	✷a	✷a	✷a	✷b	✷c	✷a	✷c	8	Low
32	Nehme (2022)[32]	✷b	✷a	✷a	✷a	✷a	✷b	✷c	✷a	b	8	Low
33	Noviello (2022)[33]	c	b	✷a	✷a	✷a	✷b	✷c	✷a	✷c	7	Low
34	Nugawela (2022)[34]<br>Stephenson (2022)[35]<br>Pereira (2023)[36]	✷a	✷a	✷a	✷a	✷a	✷b	✷c	✷a	d	8	Low
35	Rivera-Izquierdo (2022)[37]	✷b	✷a	✷a	d	NIL	✷b	✷d	✷a	✷c	7	Low
36	Robineau (2022)[38]	✷a	✷a	✷a	✷a	NIL	NIL	✷d	✷a	✷c	7	Low
37	Sandmann (2022)[39]	✷a	✷a	✷a	✷a	✷a	NIL	✷d	✷a	d	7	Low
38	Sorensen (2022)[40]<br>Spiliopoulos (2022)[41]	✷a	✷a	✷a	✷a	NIL	NIL	✷d	✷a	b	6	Moderate
39	Spatz (2022)[42]<br>Wisk (2022)[43]	c	✷a	✷a	✷a	NIL	NIL	✷c	✷a	✷c	6	Moderate
40	Strahm (2022)[44]	c	✷a	✷a	✷a	NIL	NIL	✷c	✷a	✷c	6	Moderate

	Study	1) Represen-tativeness of the COVID-19 participants (sample)	2) Selection of the non-COVID-19 participants (control)	3) Ascertainment of COVID-19 infection	4) Definition of non-COVID-19 participants	5a) Comparability of COVID-19 and non-COVID-19 participants on the basis of the design or analysis	5b) Comparability of COVID-19 and non-COVID-19 participants on the basis of the design or analysis	6) Assessment of long COVID symptoms	7) Same method of ascertainment for COVID-19 positive and negative participants	8) Non-response rate/rate of loss to follow-up	Total score	Risk of bias
41	Subramanian (2022)[45]	✷a	✷a	✷b	✷b	✷a	✷b	✷b	✷a	✷c	9	Low
42	Tartof (2022)[46]	✷a	✷a	✷b	✷b	✷a	✷b	✷b	✷a	✷c	9	Low
43	van der Maaden (2022)[47]	✷a	b	✷a	✷a	NIL	NIL	✷d	✷a	✷c	6	Moderate
44	Bernas (2023)[48]	✷a	✷a	✷a	d	NIL	NIL	✷d	✷a	b	5	Moderate
45	Merikanto (2023)[49]	✷a	✷a	✷a	✷a	NIL	NIL	✷d	✷a	e	6	Moderate
46	Miller (2023)[50]	✷a	✷a	c	c	NIL	NIL	✷d	✷a	✷a	5	Moderate
47	Mizrahi (2023)[51]	✷a	✷a	✷a	✷a	✷a	✷b	✷b	✷a	✷c	9	Low
48	Pihlaja (2023)[52]	c	✷a	✷a	c	✷a	✷b	✷c	✷a	✷c	7	Low
49	Seery (2023)[53]	c	✷a	✷a	✷a	✷a	NIL	✷d	✷a	✷c	7	Low
50	Shah (2023)[54]	✷a	✷a	✷b	✷b	✷a	✷b	✷b	✷a	✷c	9	Low
51	van Wijhe (2023)[55]	✷a	✷a	✷a	✷a	✷a	✷b	✷d	✷a	✷a	9	Low

Table S3. RRs, prevalence of symptoms in COVID-19 and non-COVID-19 participants

Symptoms	RR (95% CI)	P0 (%)	P1 (%)
**At least one symptom**	1.610 (1.489, 1.733)	25.4	40.9
**Neurologic**			
Smell	7.519 (5.826, 9.613)	1.5	11.4
Taste	5.505 (3.673, 8.141)	1.4	7.7
Brain fog	2.059 (1.355, 3.114)	1.5	3.1
Cognitive decline	1.934 (1.534, 2.431)	3	5.8
Dizziness	1.644 (1.381, 1.953)	3.2	5.3
Headache	1.366 (1.247, 1.495)	8.1	11.1
Neurological problems	1.285 (0.878, 1.876)	0.9	1.2
**General**			
Post-exertional malaise	2.867 (2.405, 3.400)	5.1	14.6
Fatigue	1.797 (1.652, 1.951)	10	18.0
Swelling of legs	1.569 (0.992, 2.478)	0.4	0.6
Fever, sweats, or chills	1.401 (1.088, 1.803)	1.2	1.7
**Respiratory**			
Shortness of breath	2.400 (2.062, 2.789)	2.7	6.5
Cough	1.417 (1.239, 1.617)	3.4	4.8
Throat pain	1.328 (1.056, 1.664)	2.9	3.9
**Dermatologic**			
Hair loss	2.022 (1.673, 2.44)	1.6	3.2
Skin rash	1.955 (1.63, 2.343)	1.6	3.1
**Cardiac**			
Chest pain	2.044 (1.285, 3.229)	1.7	3.5
Palpitations	1.270 (1.115, 1.447)	1.6	2.0
**Eye**			
Vision	1.761 (0.782, 3.933)	0.6	1.1
Itchy eyes	1.548 (1.230, 1.944)	1.6	2.5
**Ear**			
Hearing	1.488 (1.089, 2.028)	1.6	2.4
**Musculoskeletal**			
Muscle pain	1.450 (1.249, 1.679)	5.6	8.1
Joint pain	1.366 (1.203, 1.547)	5.6	7.7
Back pain	0.874 (0.634, 1.191)	13.8	12.1
**Psychiatric**			
Sleep problems	1.401 (1.109, 1.758)	8.7	12.2
Anxiety	1.212 (1.041, 1.408)	3.1	3.8
Depression	1.136 (1.003, 1.286)	3.9	4.4
**Gastrointestinal**			
Gastrointestinal symptoms	1.249 (1.079, 1.444)	2.2	2.7
Abdominal pain	1.188 (1.064, 1.327)	2.9	3.4

P0: Prevalence in non-COVID-19 participants; P1: Prevalence in COVID-19 participants; RR: Risk ratio.

Table S4. Sensitivity analysis using the data from the shortest time point

Symptoms	Studies	OR (95% CI)	I^2	tau^2
**At least one symptom**	24	2.148 (1.889, 2.443)	98.6%	0.0816
**Neurologic**				
Smell	14	8.149 (5.662, 11.729)	96.8%	0.4056
Taste	11	5.028 (3.408, 7.418)	95.7%	0.3438
Brain fog	5	2.093 (1.362, 3.218)	95.2%	0.2159
Cognitive decline	26	1.894 (1.491, 2.404)	98.3%	0.3333
Dizziness	22	1.581 (1.351, 1.850)	93.8%	0.1034
Headache	33	1.387 (1.259, 1.529)	96.2%	0.0564
Neurological problems	4	1.288 (0.877, 1.891)	78.8%	0.1098
**General**				
Post-exertional malaise	3	3.300 (2.801, 3.888)	41.0%	0.0092
Fatigue	33	1.895 (1.730, 2.077)	95.6%	0.0512
Swelling of legs	2	1.573 (0.992, 2.493)	96.1%	0.1061
Fever, sweats, or chills	17	1.373 (1.071, 1.760)	88.5%	0.1688
**Respiratory**				
Shortness of breath	35	2.447 (2.095, 2.858)	98.0%	0.1719
Cough	27	1.411 (1.232, 1.617)	96.1%	0.0921
Throat pain	14	1.401 (1.102, 1.781)	96.0%	0.1840
**Dermatologic**				
Hair loss	11	2.271 (1.435, 3.596)	97.5%	0.5014
Skin rash	12	1.237 (1.081, 1.415)	86.3%	0.0295
**Cardiac**				
Chest pain	24	1.964 (1.641, 2.351)	96.5%	0.1509
Palpitations	20	1.936 (1.613, 2.324)	95.4%	0.1374
**Eye**				
Vision	4	1.859 (0.833, 4.148)	70.8%	0.3825
Itchy eyes	2	1.562 (1.235, 1.974)	0.0%	0.0000
**Ear**				
Hearing	4	1.547 (1.449, 1.653)	0.0%	0.0000
**Musculoskeletal**				
Muscle pain	19	1.520 (1.292, 1.789)	96.2%	0.1009
Joint pain	12	1.328 (1.162, 1.517)	93.3%	0.0447
Back pain	3	0.857 (0.599, 1.228)	82.9%	0.0828
**Psychiatric**				
Sleep problems	23	1.305 (1.189, 1.434)	90.4%	0.0314
Anxiety	19	1.096 (0.980, 1.227)	91.6%	0.0390
Depression	18	1.200 (1.022, 1.409)	91.7%	0.0760
**Gastrointestinal**				
Gastrointestinal symptoms	20	1.263 (1.097, 1.453)	86.3%	0.0547
Abdominal pain	16	1.196 (1.069, 1.337)	92.6%	0.0316

Table S5. Subgroup analysis by different gender

Symptoms	Female			Male			p
	Studies	OR (95% CI)	I²	Studies	OR (95% CI)	I²	
**Neurologic**							
Cognitive decline	5	4.408 (1.983, 9.799)	99.4%	4	4.309 (1.629, 11.402)	98.9%	0.972
Dizziness	4	2.008 (1.192, 3.383)	98.5%	3	1.939 (1.053, 3.572)	97.6%	0.932
Headache	3	1.566 (0.919, 2.669)	99.5%	3	1.388 (0.815, 2.366)	98.5%	0.754
**General**							
Fatigue	4	2.473 (1.250, 4.893)	99.6%	3	2.759 (1.371, 5.550)	98.9%	0.826
**Respiratory**							
Shortness of breath	4	3.385 (1.437, 7.971)	99.5%	4	3.051 (1.188, 7.838)	99.1%	0.873
Cough	3	1.349 (0.969, 1.877)	98.2%	3	1.167 (0.965, 1.410)	89.5%	0.456
Throat pain	3	1.156 (0.877, 1.523)	93.1%	3	1.255 (0.913, 1.727)	87.8%	0.700
**Cardiac**							
Chest pain	4	2.123 (0.922, 4.890)	99.4%	4	2.392 (1.009, 5.671)	99.0%	0.846
Palpitations	3	1.626 (0.753, 3.512)	98.9%	2	1.842 (0.568, 5.979)	98.0%	0.862
**Dermatologic**							
Hair loss	3	1.897 (0.687, 5.240)	97.5%	3	1.349 (0.526, 3.457)	84.8%	0.629
Skin rash	1	0.913 (0.819, 1.018)	NA	2	1.149 (0.627, 2.104)	90.4%	0.463
**Eye**							
Vision	1	0.982 (0.831, 1.160)	NA	2	3.083 (0.168, 56.625)	81.3%	0.442
**Musculoskeletal**							
Muscle pain	3	1.327 (0.763, 2.309)	98.7%	3	1.281 (0.712, 2.306)	97.5%	0.931
Joint pain	2	1.453 (0.907, 2.328)	98.0%	2	1.337 (0.715, 2.500)	96.9%	0.836
**Psychiatric**							
Sleep problems	4	2.275 (0.987, 5.241)	99.6%	4	2.189 (1.001, 4.790)	98.4%	0.948

Anxiety	4	1.334 (0.959, 1.855)	96.7%	4	1.227 (0.964, 1.563)	83.9%	0.691
Depression	4	1.291 (1.039, 1.604)	90.0%	3	1.330 (1.103, 1.605)	74.1%	0.838
**Gastrointestinal**							
Gastrointestinal symptoms	3	1.304 (1.073, 1.585)	66.2%	3	1.178 (0.810, 1.713)	75.1%	0.637
Abdominal pain	3	1.244 (0.889, 1.742)	97.7%	3	1.102 (0.776, 1.566)	92.1%	0.626

*p<0.05. NA: Not available.

Table S6. Subgroup analysis by risk of bias

Symptoms	Low risk of bias			Moderate risk of bias			P
	Studies	OR (95% CI)	I²	Studies	OR (95% CI)	I²	
**At least one symptom**	10	1.953 (1.768, 2.156)	96.1%	14	1.890 (1.394, 2.563)	97.3%	0.842
**Neurologic**							
Smell	7	6.930 (4.514, 10.639)	95.7%	7	11.254 (8.276, 15.304)	72.8%	0.072
Taste	6	4.371 (2.312, 8.262)	96.7%	5	8.661 (5.331, 14.071)	76.8%	0.094
Brain fog	3	1.601 (0.878, 2.921)	88.7%	2	2.939 (2.667, 3.240)	0.0%	0.051
Cognitive decline	19	1.931 (1.557, 2.395)	94.2%	7	2.017 (1.044, 3.897)	99.2%	0.902
Dizziness	14	1.716 (1.449, 2.033)	88.0%	8	1.472 (1.013, 2.139)	95.4%	0.463
Headache	22	1.365 (1.230, 1.515)	94.9%	10	1.504 (1.137, 1.990)	95.4%	0.523
Neurological problems	2	1.603 (1.336, 1.923)	0.0%	2	1.121 (0.538, 2.336)	89.0%	0.354
**General**							
Post-exertional malaise	1	2.640 (1.650, 4.224)	NA	2	3.282 (2.606, 4.132)	68.3%	0.415
Fatigue	23	2.092 (1.844, 2.374)	95.8%	10	1.724 (1.384, 2.146)	95.6%	0.133
Fever, sweats, or chills	11	1.587 (1.237, 2.036)	74.1%	6	1.178 (0.656, 2.114)	95.2%	0.358
**Respiratory**							
Shortness of breath	24	2.489 (2.108, 2.938)	97.6%	11	2.459 (1.539, 3.929)	97.7%	0.962
Cough	20	1.577 (1.356, 1.834)	95.6%	7	1.099 (0.715, 1.689)	96.8%	0.120
Throat pain	8	1.477 (1.162, 1.878)	91.1%	6	1.201 (0.713, 2.024)	97.0%	0.480
**Cardiac**							
Chest pain	17	1.719 (1.425, 2.074)	95.1%	7	2.901 (1.665, 5.054)	97.0%	0.080
Palpitations	12	1.856 (1.479, 2.330)	94.5%	8	2.172 (1.568, 3.009)	93.4%	0.438
**Dermatologic**							
Hair loss	7	2.008 (0.990, 4.074)	98.4%	4	2.392 (1.762, 3.246)	61.8%	0.657
Skin rash	9	1.217 (1.076, 1.377)	81.2%	3	2.332 (0.971, 5.599)	92.0%	0.149

**Eye**							
Vision	3	1.225 (0.731, 2.053)	38.5%	1	1.225 (0.731, 2.053)	0.0%	0.041*
**Ear**							
Hearing	3	1.475 (1.155, 1.882)	18.2%	1	3.748 (0.973, 14.442)	NA	0.182
**Musculoskeletal**							
Joint pain	8	1.217 (1.123, 1.318)	63.6%	4	1.926 (1.628, 2.279)	38.9%	<0.001*
Muscle pain	15	1.528 (1.345, 1.736)	90.7%	4	1.457 (0.649, 3.273)	99.0%	0.910
**Psychiatric**							
Sleep problems	14	1.245 (1.088, 1.425)	88.5%	9	1.627 (1.008, 2.627)	99.5%	0.292
Anxiety	14	1.016 (0.892, 1.156)	88.2%	5	1.359 (1.116, 1.654)	93.5%	0.015*
Depression	12	1.059 (0.960, 1.169)	16.2%	6	1.424 (1.187, 1.709)	91.9%	0.005*
**Gastrointestinal**							
Gastrointestinal symptoms	14	1.139 (0.961, 1.350)	83.5%	6	1.585 (1.286, 1.953)	61.0%	0.016*
Abdominal pain	10	1.090 (0.970, 1.225)	92.2%	6	1.454 (1.120, 1.886)	83.5%	0.048*

*p<0.05. NA: Not available.

Table S7. Subgroup analysis by study design

Symptoms	Cohort study			Cross-sectional study			p
	Studies	OR (95% CI)	I²	Studies	OR (95% CI)	I²	
**At least one symptom**	22	2.021 (1.836, 2.225)	96.0%	2	1.020 (0.13, 7.992)	96.5%	0.515
**Neurologic**							
Smell	11	7.380 (5.022, 10.846)	93.0%	3	12.790 (11.271, 14.513)	18.3%	0.008*
Taste	9	4.831 (2.917, 8.003)	92.4%	2	11.963 (10.297, 13.900)	0.0%	0.001*
Brain fog	4	1.901 (1.095, 3.300)	93.5%	1	2.913 (2.618, 3.242)	NA	0.137
Cognitive decline	20	1.883 (1.483, 2.391)	96.0%	6	2.207 (1.541, 3.159)	96.6%	0.471
Dizziness	16	1.413 (1.157, 1.725)	91.5%	6	2.292 (1.977, 2.657)	73.3%	<0.001*
Headache	27	1.262 (1.145, 1.390)	93.9%	6	2.007 (1.772, 2.274)	72.1%	<0.001*
**General**							
Post-exertional malaise	2	2.797 (2.259, 3.464)	0.0%	1	3.611 (3.229, 4.039)	NA	0.038*
Fatigue	27	1.936 (1.721, 2.177)	95.3%	6	2.075 (1.563, 2.755)	97.2%	0.657
Fever, sweats, or chills	13	1.143 (0.870, 1.501)	80.4%	4	2.260 (1.257, 4.065)	95.4%	0.039*
**Respiratory**							
Shortness of breath	29	2.192 (1.873, 2.565)	97.3%	6	4.012 (3.050, 5.277)	90.5%	<0.001*
Cough	22	1.287 (1.108, 1.495)	95.5%	5	2.061 (1.349, 3.148)	96.6%	0.040*
Throat pain	10	1.091 (0.845, 1.409)	94.1%	4	2.272 (1.175, 4.392)	96.3%	0.042*
**Cardiac**							
Chest pain	20	1.814 (1.525, 2.157)	94.8%	4	3.489 (1.659, 7.335)	96.9%	0.093
Palpitations	16	1.887 (1.544, 2.307)	93.6%	4	2.352 (1.619, 3.416)	93.4%	0.309
**Dermatologic**							
Skin rash	11	1.297 (1.125, 1.495)	85.3%	1	1.111 (0.819, 1.507)	NA	0.367
**Musculoskeletal**							
Joint pain	11	1.307 (1.178, 1.450)	78.7%	1	1.878 (1.744, 2.022)	NA	<0.001*

Symptoms	Cohort study			Cross-sectional study			p
	Studies	OR (95% CI)	I²	Studies	OR (95% CI)	I²	
Muscle pain	16	1.433 (1.220, 1.682)	94.0%	3	1.489 (1.167, 1.901)	99.0%	0.628
**Psychiatric**							
Sleep problems	20	1.304 (1.149, 1.480)	91.2%	3	2.045 (0.902, 4.633)	99.8%	0.287
Anxiety	17	1.093 (0.953, 1.253)	91.9%	2	1.426 (1.105, 1.840)	96.8%	0.072
Depression	16	1.172 (0.944, 1.456)	91.6%	2	1.498 (1.422, 1.578)	10.0%	0.031*
**Gastrointestinal**							
Gastrointestinal symptoms	16	1.122 (0.949, 1.326)	81.0%	4	1.618 (1.401, 1.869)	56.6%	0.001*
Abdominal pain	13	1.086 (0.972, 1.213)	90.4%	3	1.646 (1.418, 1.912)	55.2%	<0.001*

*p<0.05. NA: Not available.

Table S8. Subgroup analysis by adjustment for confounders

Symptoms	Adjusted results			Crude results			p
	Studies	OR (95% CI)	I^2	Studies	OR (95% CI)	I^2	
**At least one symptom**	3	1.305 (1.003, 1.699)	86.9%	26	2.122 (1.860, 2.421)	98.5%	0.001*
**Neurologic**							
Smell	4	5.401 (2.816, 10.357)	97.7%	14	8.499 (6.350, 11.374)	93.8%	0.213
Taste	4	3.874 (1.664, 9.018)	98.0%	11	6.656 (4.188, 10.58)	96.1%	0.271
Brain fog	2	2.047 (0.892, 4.695)	93.0%	5	2.071 (1.353, 3.171)	95.1%	0.980
Cognitive decline	9	1.817 (1.222, 2.702)	95.7%	24	2.037 (1.582, 2.624)	97.9%	0.633
Dizziness	8	1.534 (1.278, 1.840)	85.3%	20	1.743 (1.417, 2.144)	95.3%	0.363
Headache	12	1.279 (1.120, 1.459)	95.8%	29	1.476 (1.315, 1.657)	96.3%	0.110
Neurological problems	2	1.603 (1.336, 1.923)	0.0%	3	1.233 (0.691, 2.200)	79.7%	0.397
**General**							
Post-exertional malaise	1	2.640 (1.65, 4.224)	68.3%	2	3.282 (2.606, 4.132)	NA	0.415
Fatigue	10	2.242 (1.792, 2.806)	97.3%	29	1.932 (1.759, 2.123)	94.5%	0.230
Swelling of legs	2	1.573 (0.992, 2.493)	96.1%	1	1.099 (1.009, 1.197)	NA	0.134
Fever, sweats, or chills	5	1.651 (1.485, 1.837)	3.5%	14	1.440 (1.059, 1.956)	90.8%	0.407
**Respiratory**							
Shortness of breath	11	2.612 (1.921, 3.552)	99.0%	31	2.552 (2.125, 3.065)	98.1%	0.899
Cough	11	1.572 (1.284, 1.925)	97.5%	23	1.505 (1.298, 1.745)	95.6%	0.733
Throat pain	3	1.214 (0.940, 1.569)	91.1%	13	1.313 (1.028, 1.676)	94.9%	0.666
**Cardiac**							
Chest pain	10	1.603 (1.264, 2.033)	97.0%	19	2.272 (1.806, 2.858)	97.4%	0.039*
Palpitations	7	1.963 (1.600, 2.408)	97.1%	18	1.963 (1.600, 2.408)	95.5%	0.831
**Dermatologic**							
Hair loss	6	1.840 (0.853, 3.968)	98.7%	9	2.051 (1.076, 3.907)	98.4%	0.832

Skin rash	6	1.245 (1.079, 1.437)	89.3%	9	1.272 (1.057, 1.531)	91.0%	0.855
**Eye**							
Vision	3	1.225 (0.731, 2.053)	38.5%	3	2.107 (0.545, 8.147)	68.7%	0.463
Itchy eyes	1	1.680 (1.011, 2.792)	NA	2	1.554 (1.229, 1.965)	0.0%	0.785
**Ear**							
Hearing	3	1.492 (1.094, 2.034)	29.1%	3	1.551 (1.451, 1.657)	0.0%	0.810
**Musculoskeletal**							
Muscle pain	9	1.436 (1.165, 1.769)	94.7%	17	1.546 (1.297, 1.842)	96.8%	0.594
Joint pain	7	1.220 (1.096, 1.358)	80.7%	10	1.404 (1.191, 1.654)	93.8%	0.162
Back pain	2	0.726 (0.586, 0.899)	62.6%	3	0.979 (0.755, 1.270)	0.0%	0.081
**Psychiatric**							
Sleep problems	10	1.155 (0.996, 1.340)	91.7%	22	1.512 (1.156, 1.977)	99.3%	0.086
Anxiety	8	0.976 (0.860, 1.107)	87.4%	16	0.976 (0.860, 1.107)	95.2%	0.026*
Depression	6	1.081 (1.022, 1.144)	4.4%	17	1.418 (1.190, 1.688)	95.4%	0.004*
**Respiratory**							
Shortness of breath	11	2.612 (1.921, 3.552)	99.0%	31	2.552 (2.125, 3.065)	98.1%	0.899
Cough	11	1.572 (1.284, 1.925)	97.5%	23	1.505 (1.298, 1.745)	95.6%	0.733
Throat pain	3	1.214 (0.940, 1.569)	91.1%	13	1.313 (1.028, 1.676)	94.9%	0.666
**Gastrointestinal**							
Gastrointestinal symptoms	9	1.244 (1.034, 1.498)	86.8%	18	1.260 (1.078, 1.472)	85.6%	0.921
Abdominal pain	3	1.088 (0.964, 1.228)	94.7%	14	1.189 (1.029, 1.373)	94.0%	0.358

*p<0.05. NA: Not available.

Figure S1. Forest plots of the meta-analysis of each symptom

At least one symptom

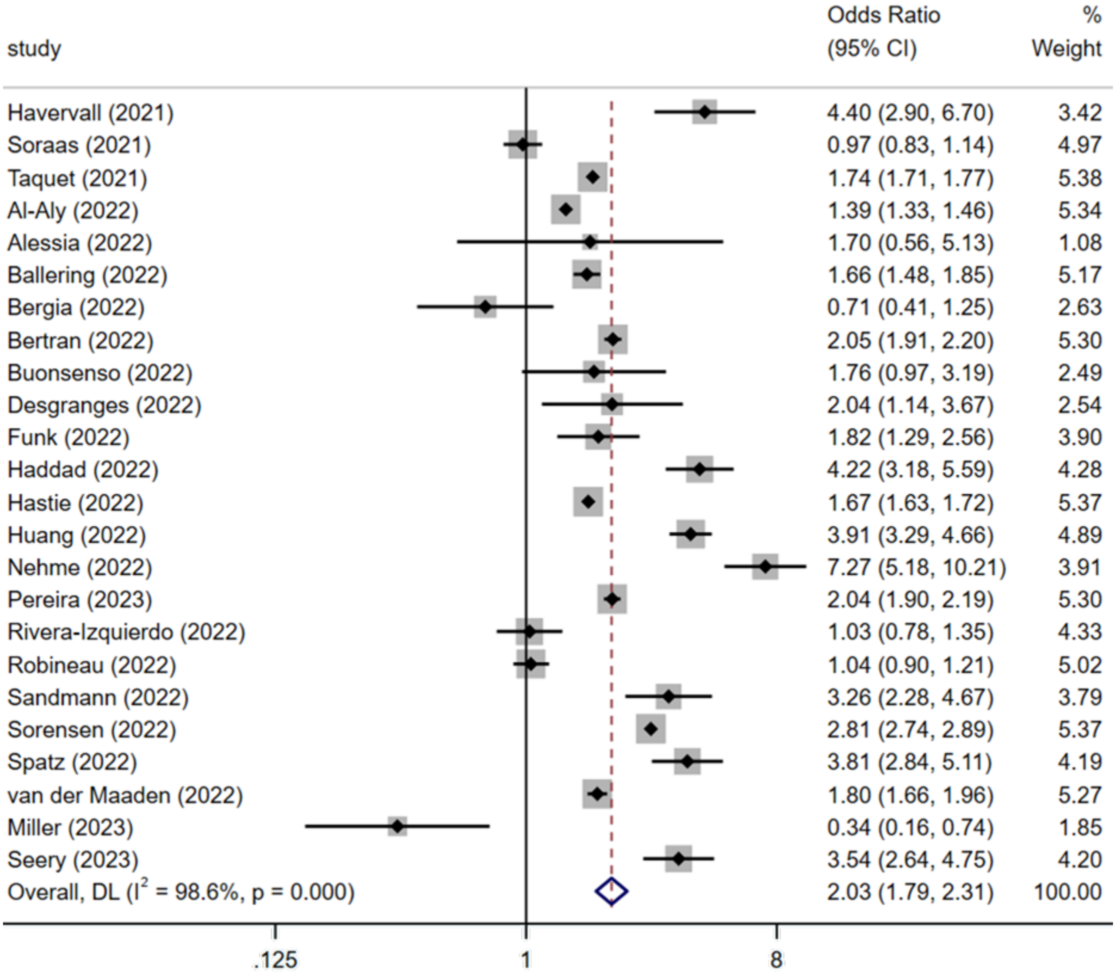

Smell

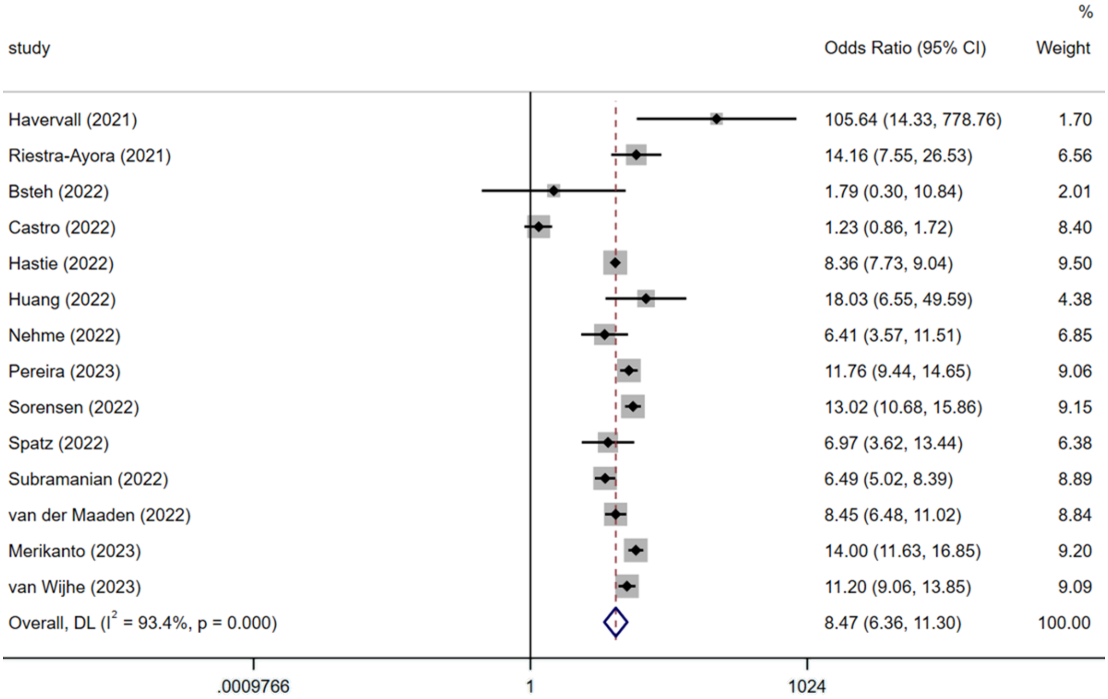

Taste

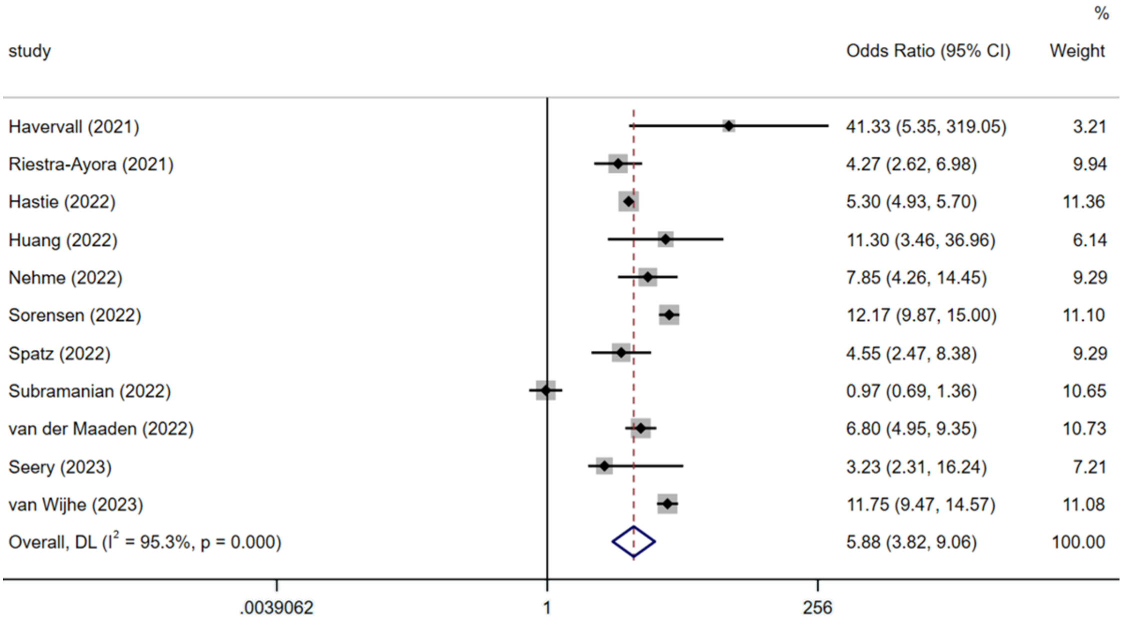

Brain fog

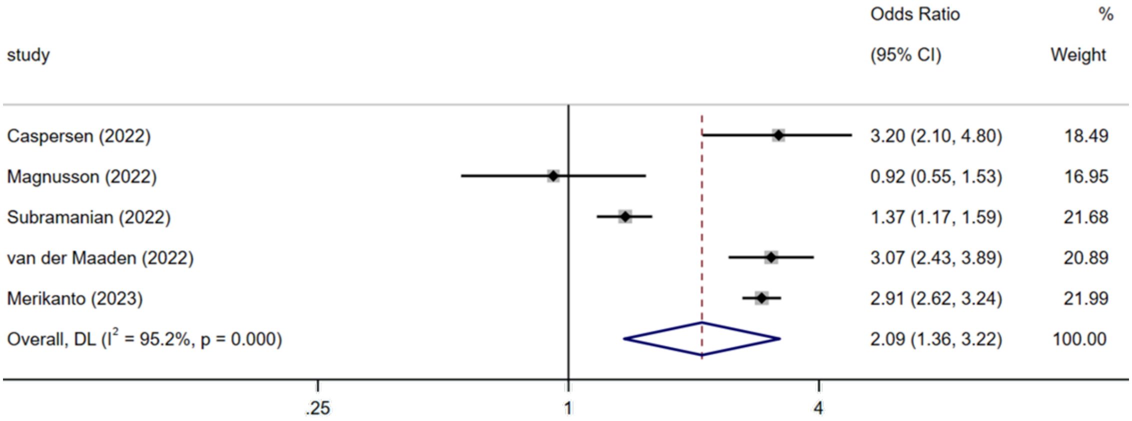

Cognitive decline

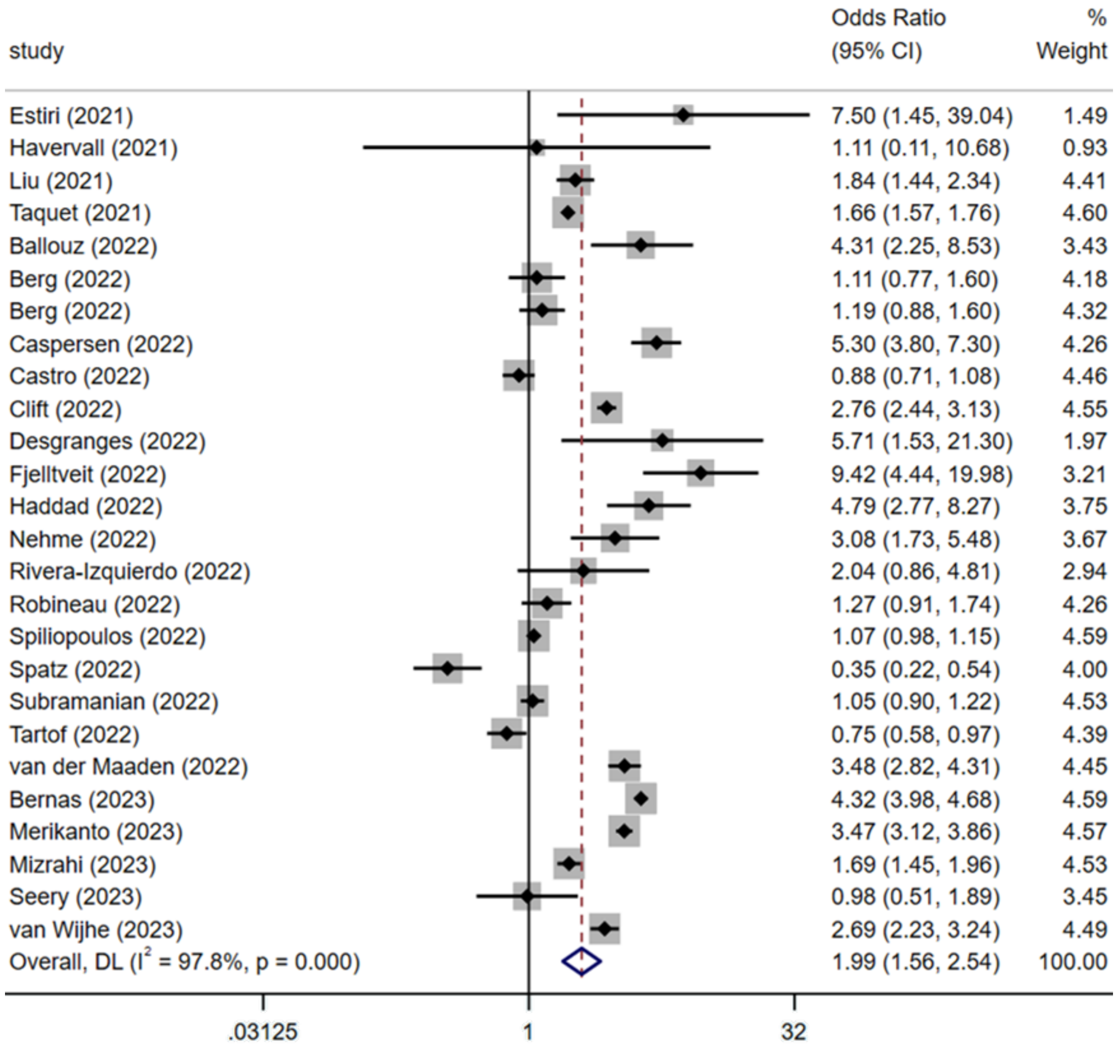

Dizziness

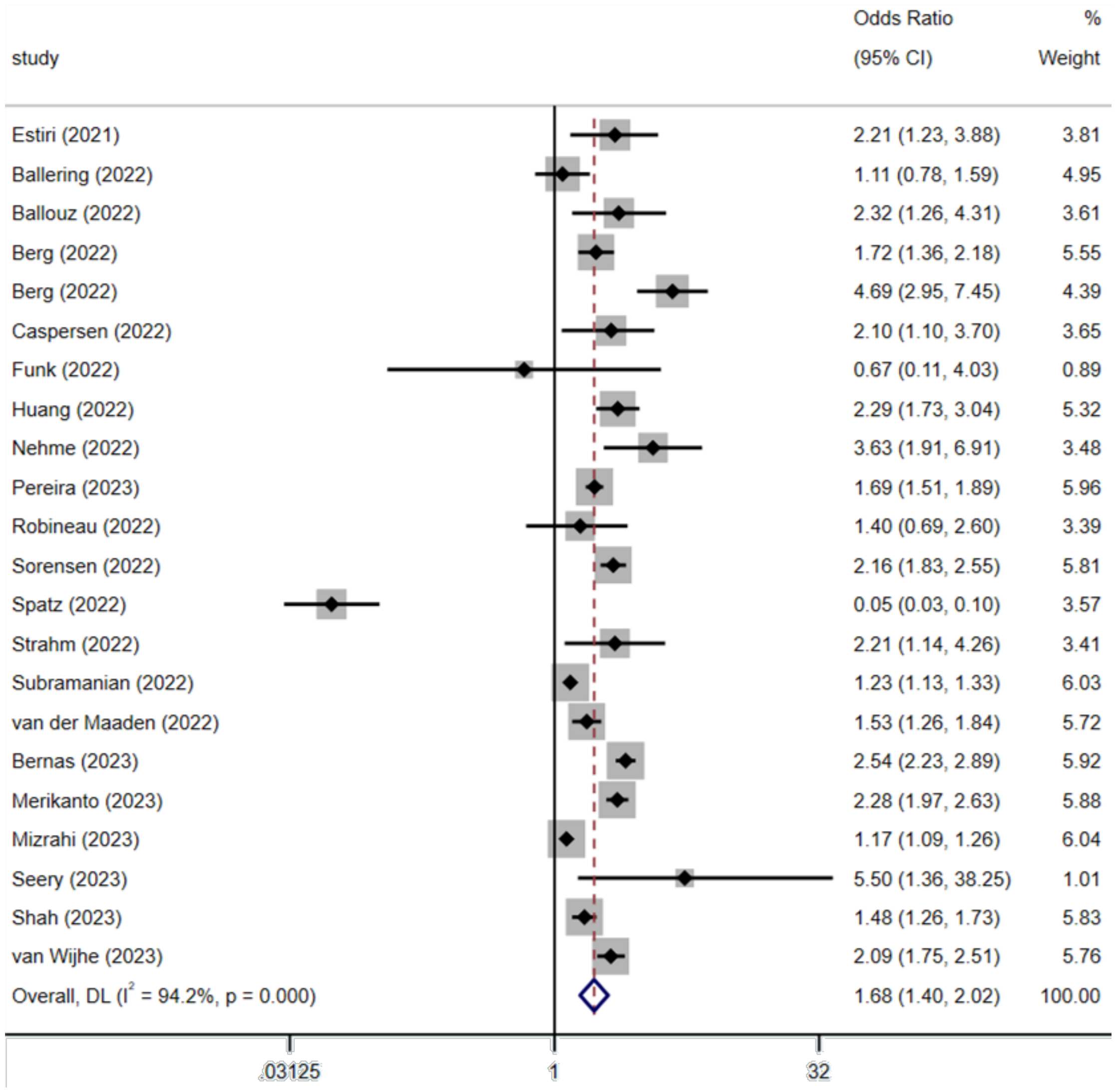
study
Odds Ratio
(95% CI)
%
Weight
Estiri (2021) 2.21 (1.23, 3.88) 3.81
Ballering (2022) 1.11 (0.78, 1.59) 4.95
Ballouz (2022) 2.32 (1.26, 4.31) 3.61
Berg (2022) 1.72 (1.36, 2.18) 5.55
Berg (2022) 4.69 (2.95, 7.45) 4.39
Caspersen (2022) 2.10 (1.10, 3.70) 3.65
Funk (2022) 0.67 (0.11, 4.03) 0.89
Huang (2022) 2.29 (1.73, 3.04) 5.32
Nehme (2022) 3.63 (1.91, 6.91) 3.48
Pereira (2023) 1.69 (1.51, 1.89) 5.96
Robineau (2022) 1.40 (0.69, 2.60) 3.39
Sorensen (2022) 2.16 (1.83, 2.55) 5.81
Spatz (2022) 0.05 (0.03, 0.10) 3.57
Strahm (2022) 2.21 (1.14, 4.26) 3.41
Subramanian (2022) 1.23 (1.13, 1.33) 6.03
van der Maaden (2022) 1.53 (1.26, 1.84) 5.72
Bernas (2023) 2.54 (2.23, 2.89) 5.92
Merikanto (2023) 2.28 (1.97, 2.63) 5.88
Mizrahi (2023) 1.17 (1.09, 1.26) 6.04
Seery (2023) 5.50 (1.36, 38.25) 1.01
Shah (2023) 1.48 (1.26, 1.73) 5.83
van Wijhe (2023) 2.09 (1.75, 2.51) 5.76
Overall, DL (I² = 94.2%, p = 0.000) 1.68 (1.40, 2.02) 100.00
.03125
1
32

Headache

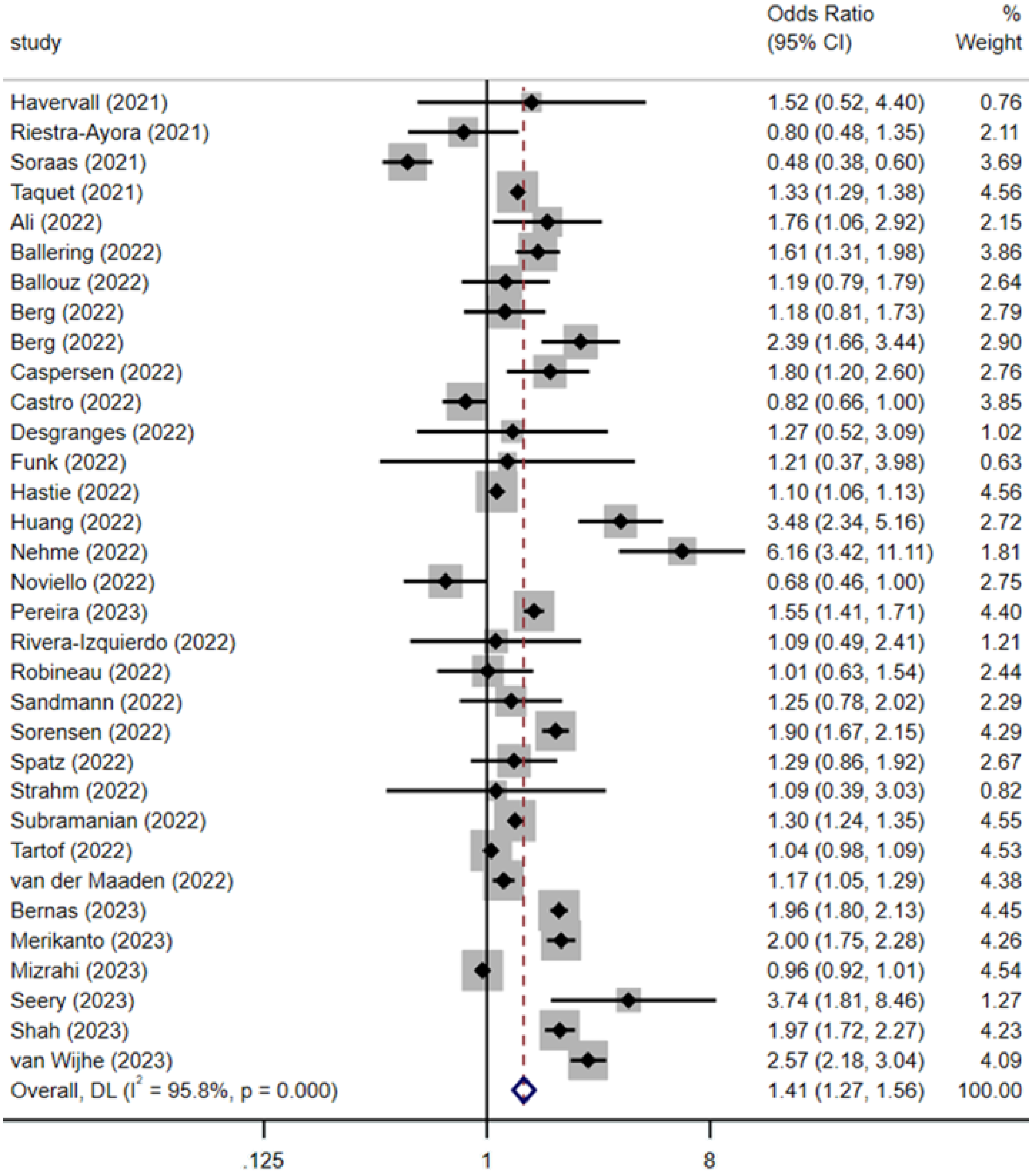

Neurological problems

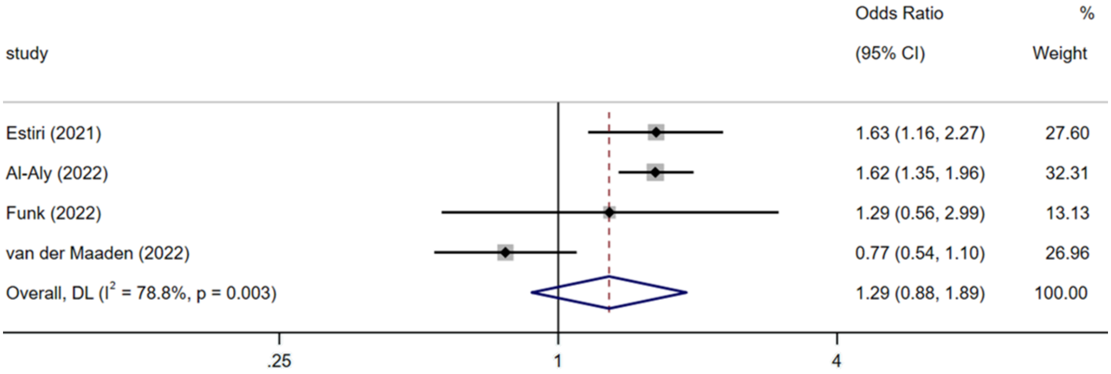

Post-exertional malaise

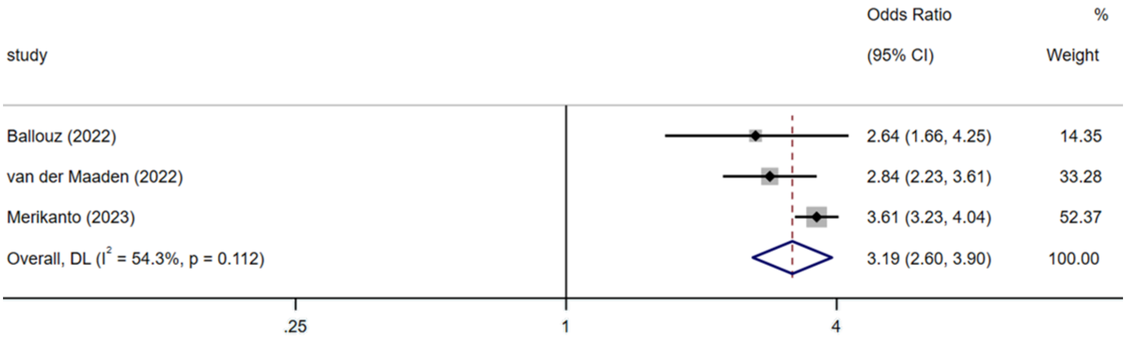

Fatigue

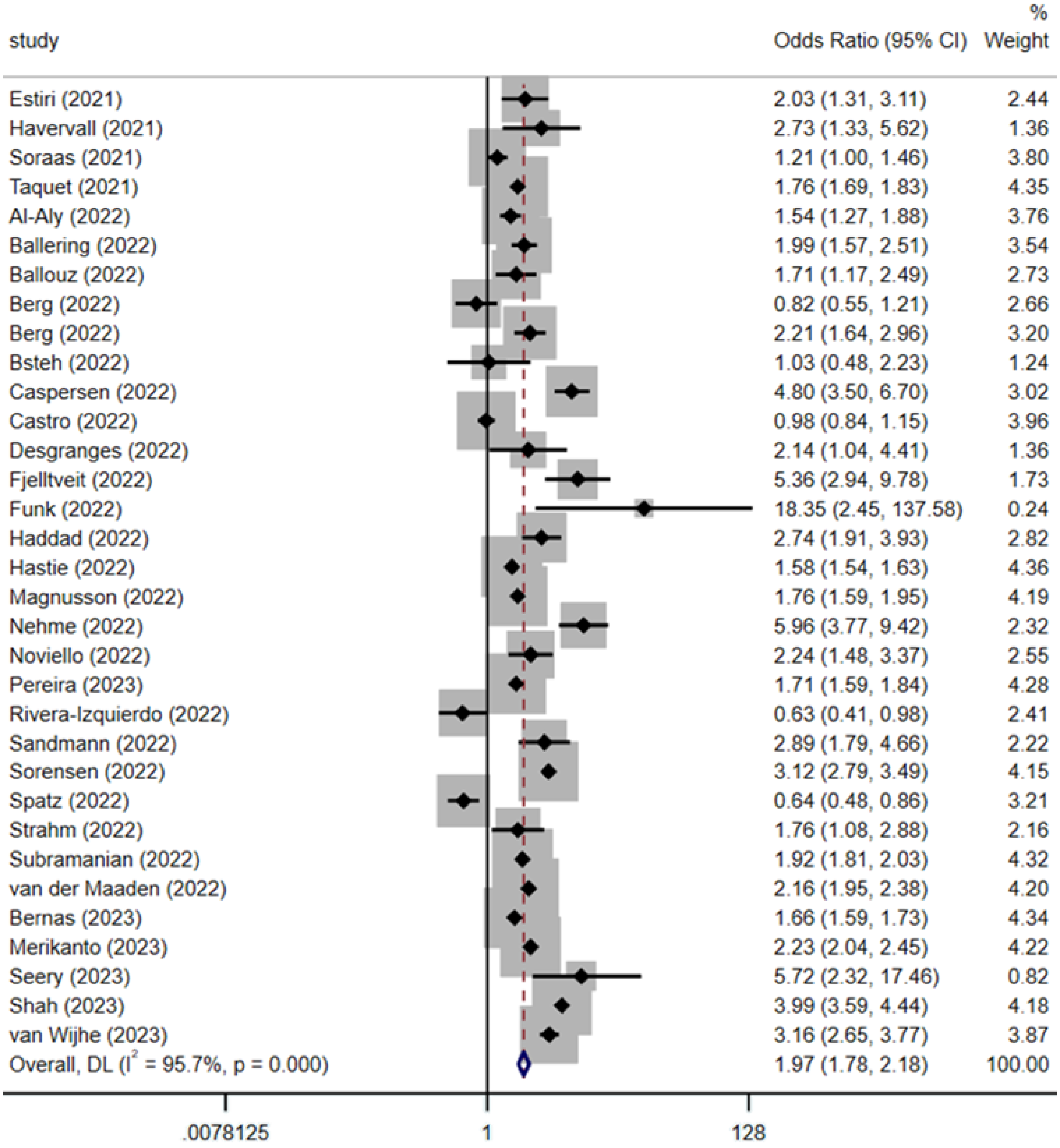

Swelling of legs

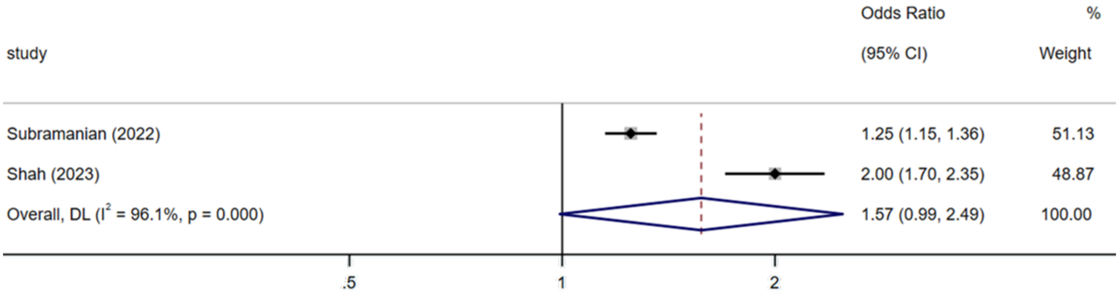

Fever, sweats, or chills

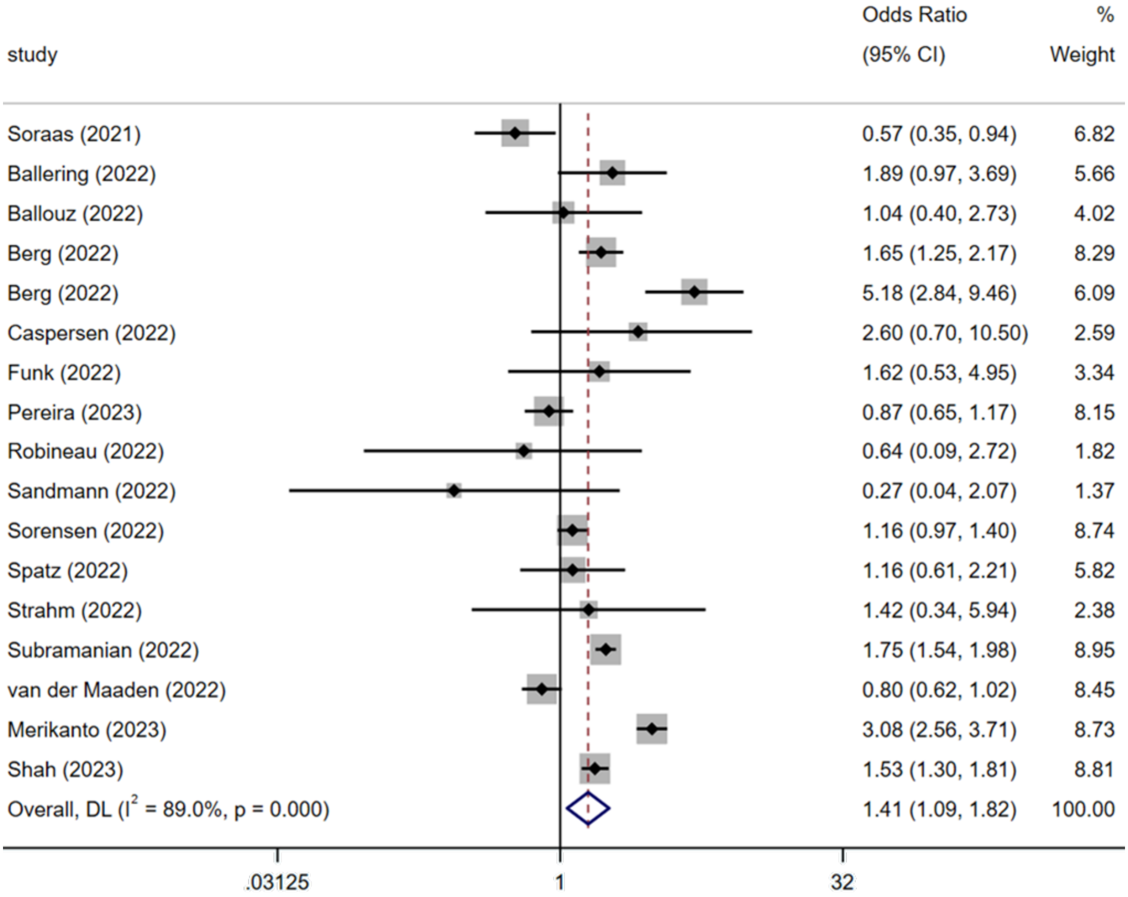

Shortness of breath

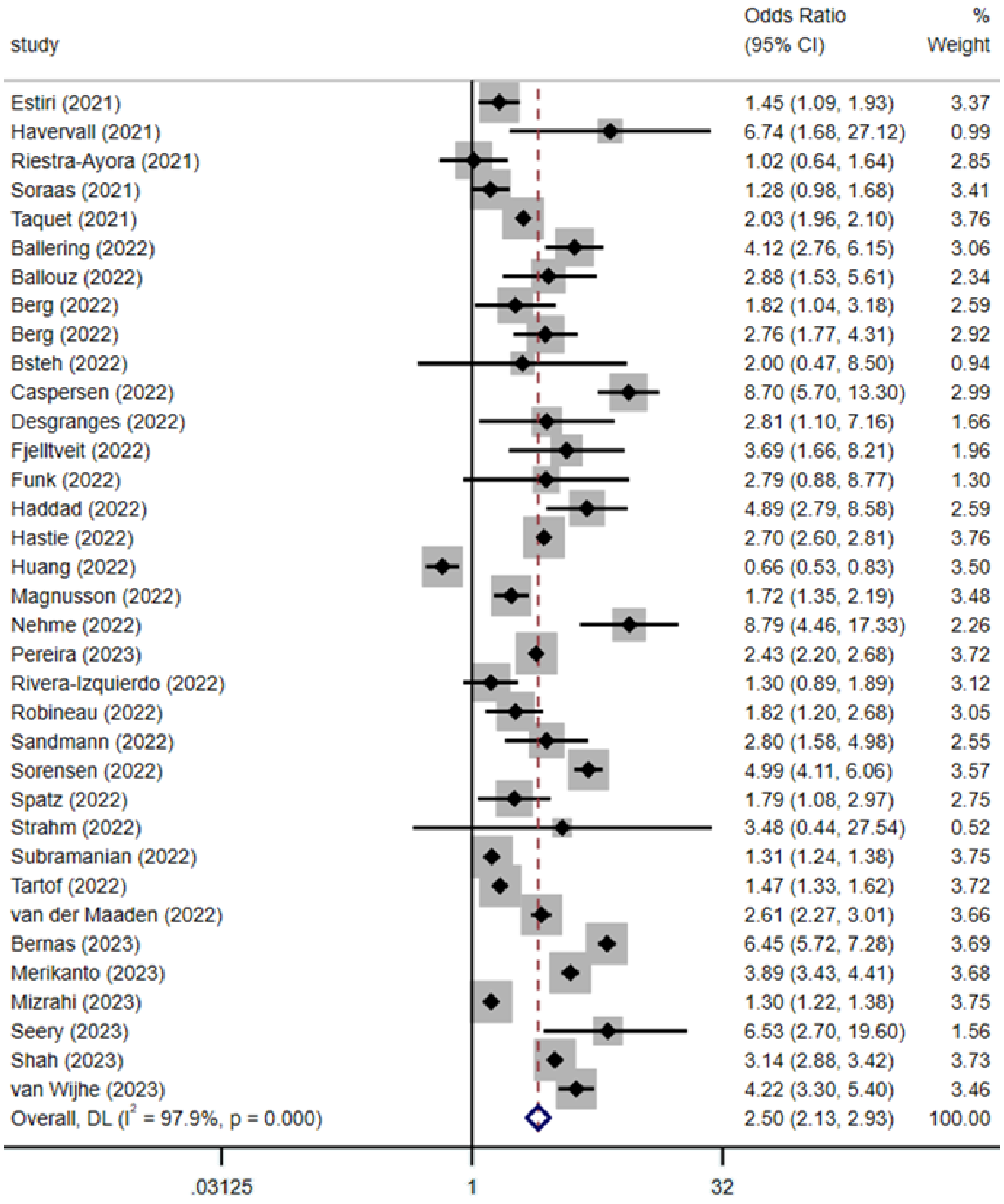

Cough

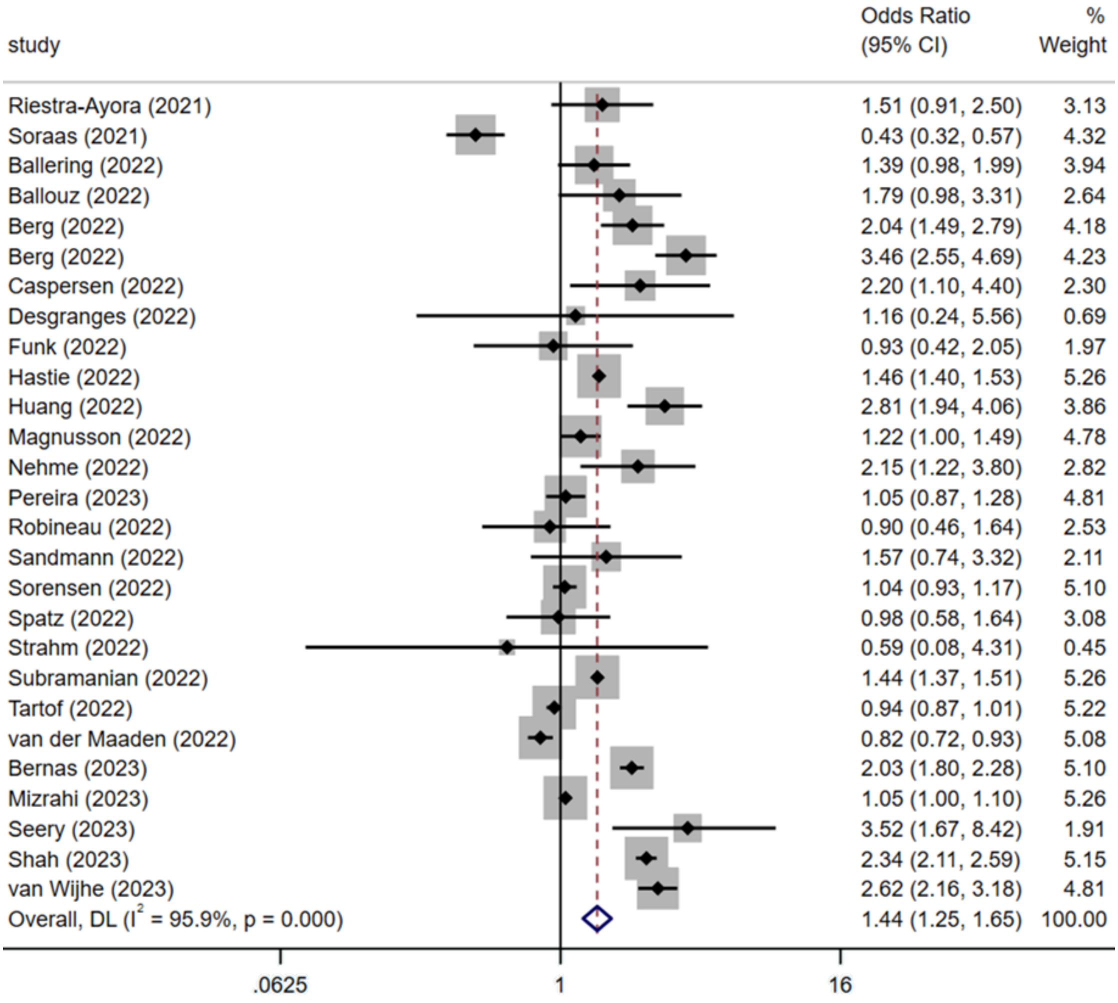

Throat pain

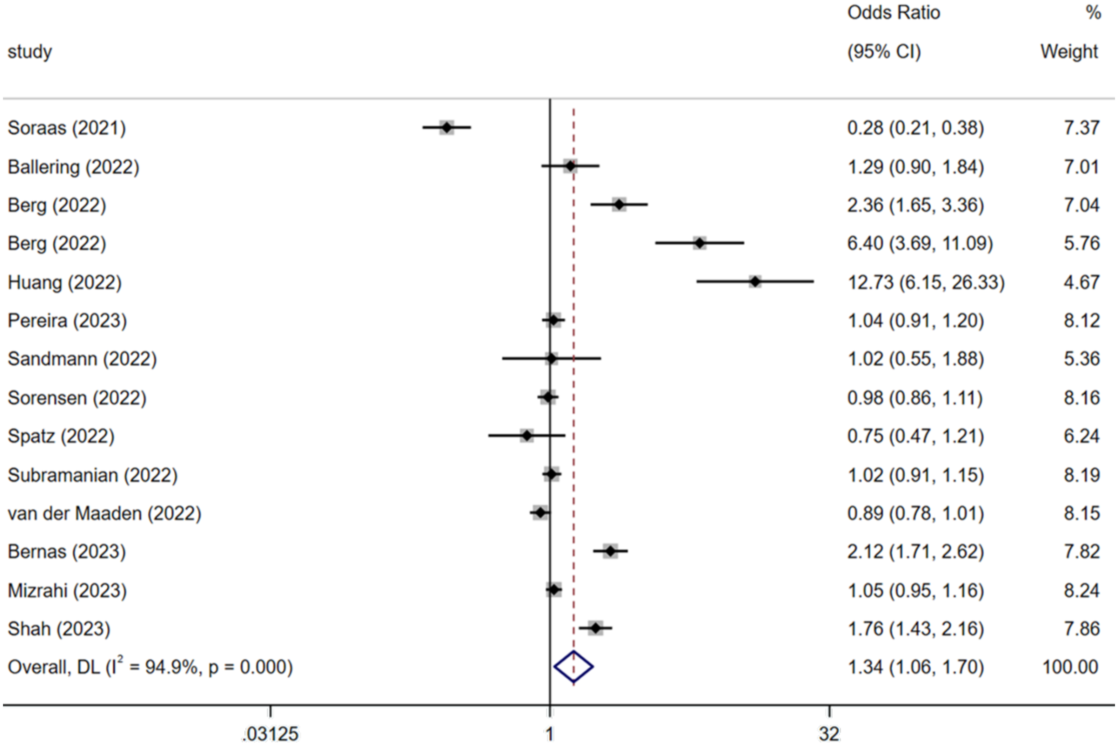

Chest pain

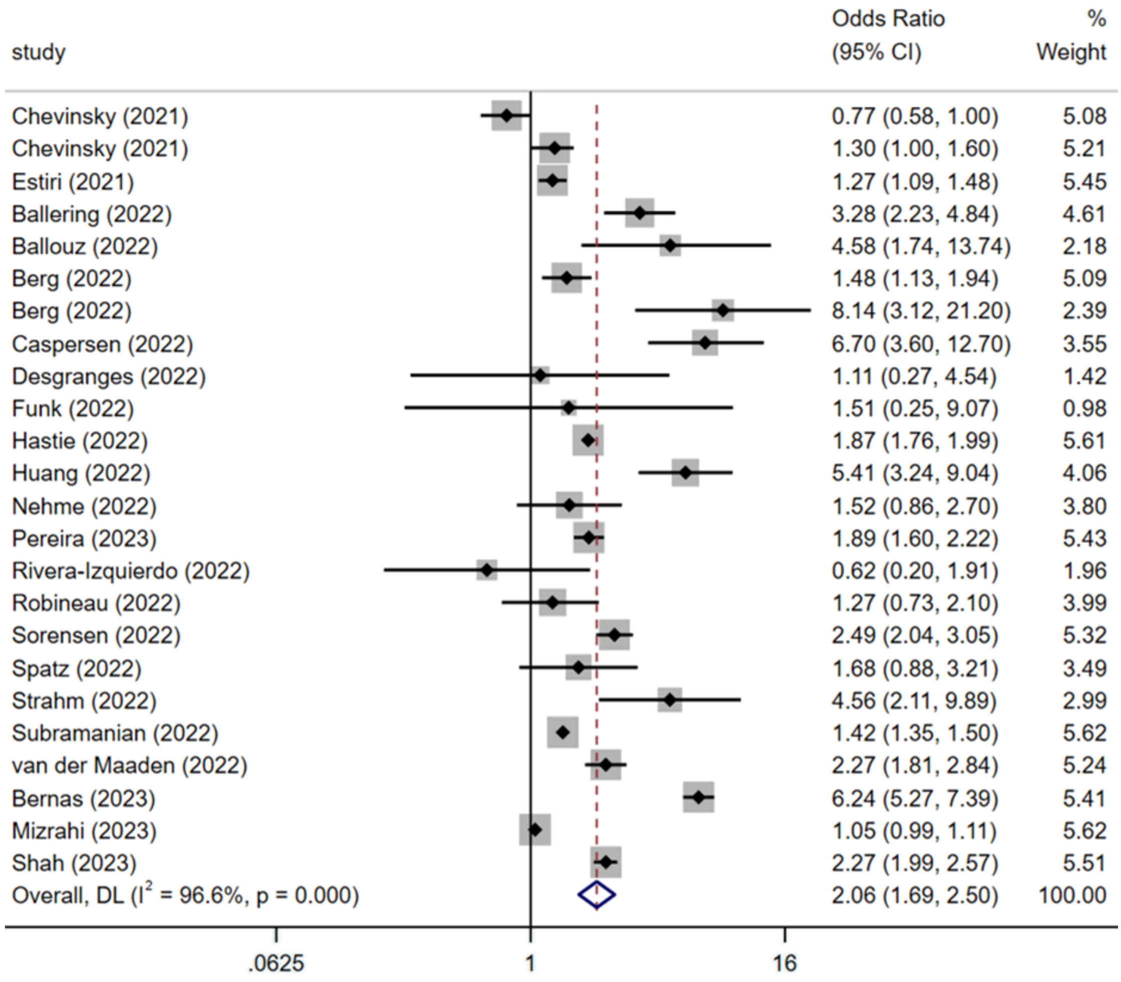

study
Odds Ratio (95% CI)
% Weight
Chevinsky (2021) 0.77 (0.58, 1.00) 5.08
Chevinsky (2021) 1.30 (1.00, 1.60) 5.21
Estiri (2021) 1.27 (1.09, 1.48) 5.45
Ballering (2022) 3.28 (2.23, 4.84) 4.61
Ballouz (2022) 4.58 (1.74, 13.74) 2.18
Berg (2022) 1.48 (1.13, 1.94) 5.09
Berg (2022) 8.14 (3.12, 21.20) 2.39
Caspersen (2022) 6.70 (3.60, 12.70) 3.55
Desgranges (2022) 1.11 (0.27, 4.54) 1.42
Funk (2022) 1.51 (0.25, 9.07) 0.98
Hastie (2022) 1.87 (1.76, 1.99) 5.61
Huang (2022) 5.41 (3.24, 9.04) 4.06
Nehme (2022) 1.52 (0.86, 2.70) 3.80
Pereira (2023) 1.89 (1.60, 2.22) 5.43
Rivera-Izquierdo (2022) 0.62 (0.20, 1.91) 1.96
Robineau (2022) 1.27 (0.73, 2.10) 3.99
Sorensen (2022) 2.49 (2.04, 3.05) 5.32
Spatz (2022) 1.68 (0.88, 3.21) 3.49
Strahm (2022) 4.56 (2.11, 9.89) 2.99
Subramanian (2022) 1.42 (1.35, 1.50) 5.62
van der Maaden (2022) 2.27 (1.81, 2.84) 5.24
Bernas (2023) 6.24 (5.27, 7.39) 5.41
Mizrahi (2023) 1.05 (0.99, 1.11) 5.62
Shah (2023) 2.27 (1.99, 2.57) 5.51
Overall, DL (I² = 96.6%, p = 0.000) 2.06 (1.69, 2.50) 100.00
.0625
1
16

Palpitations

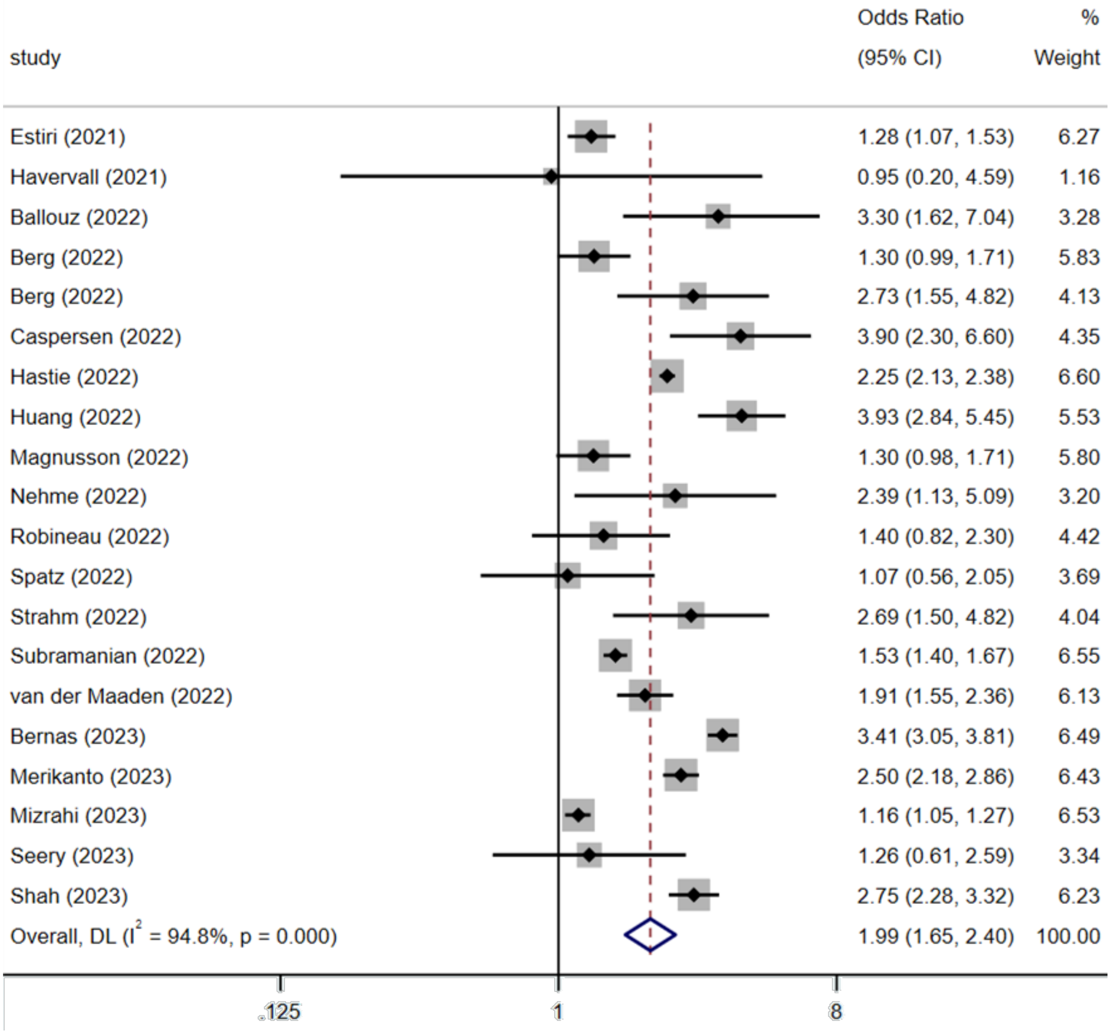

Hair loss

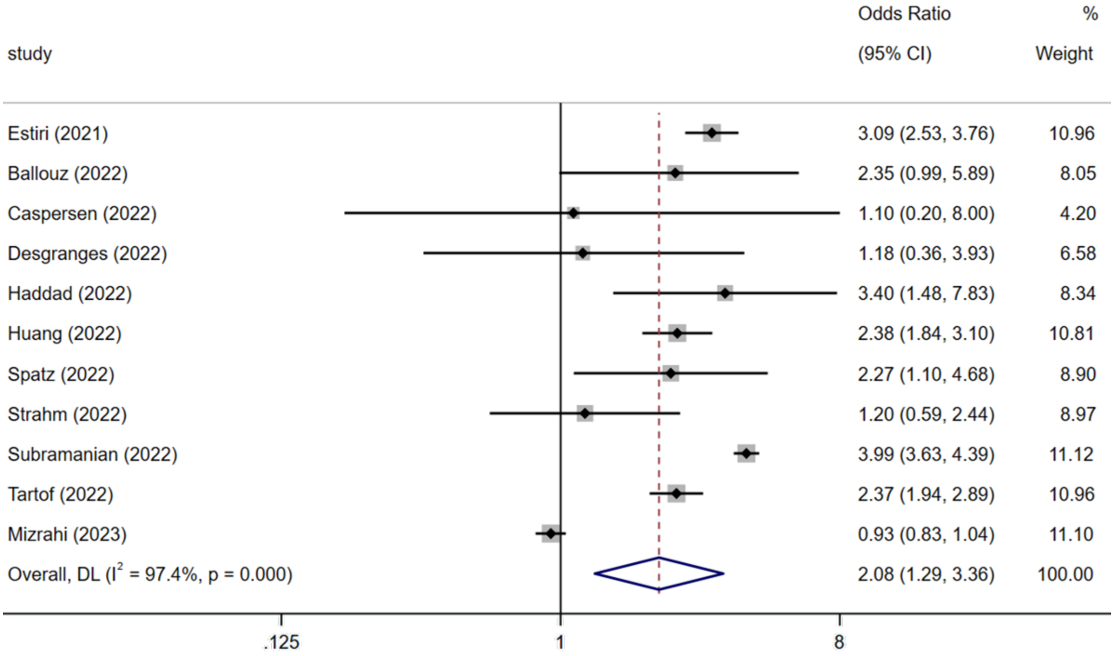

Skin rash

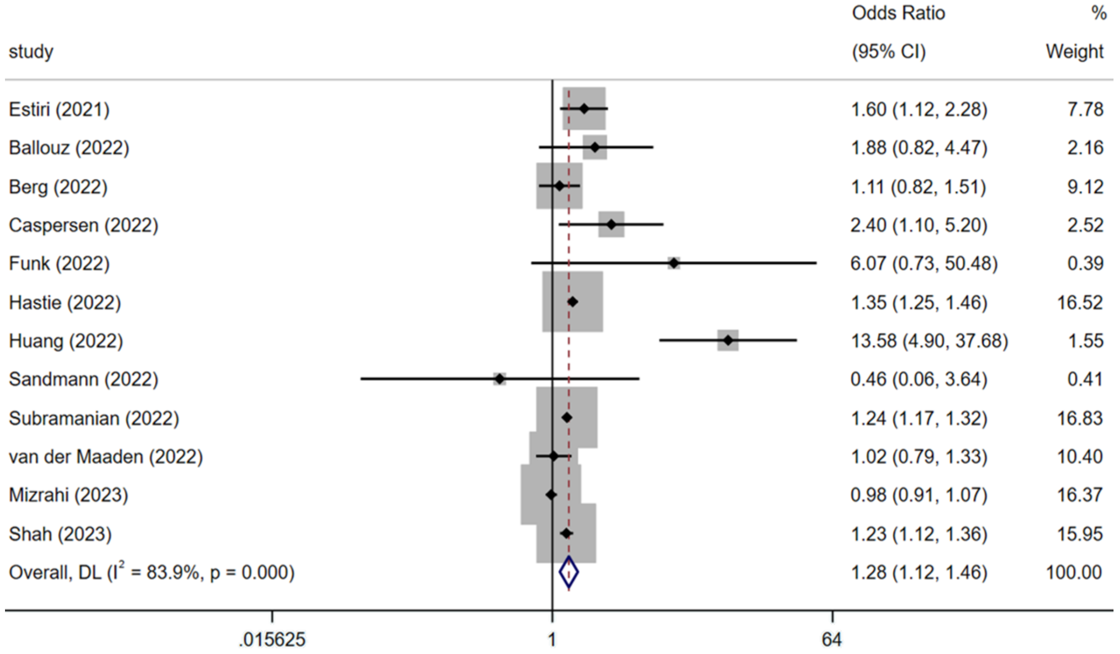

Vision

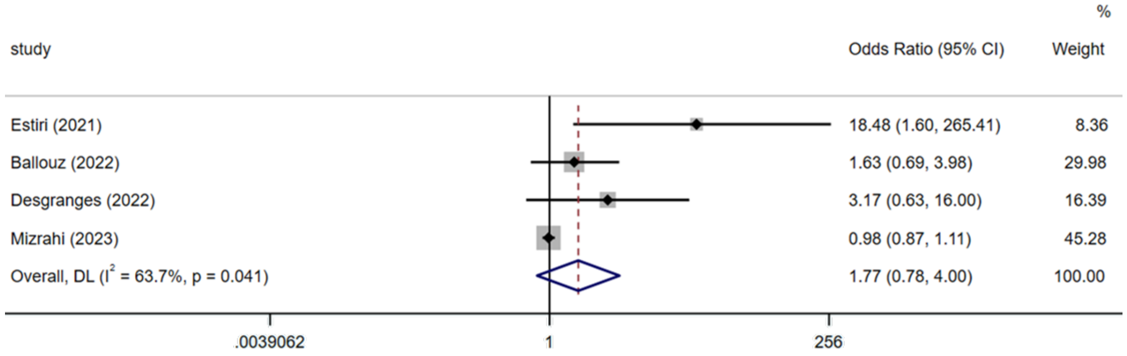

Itchy eyes

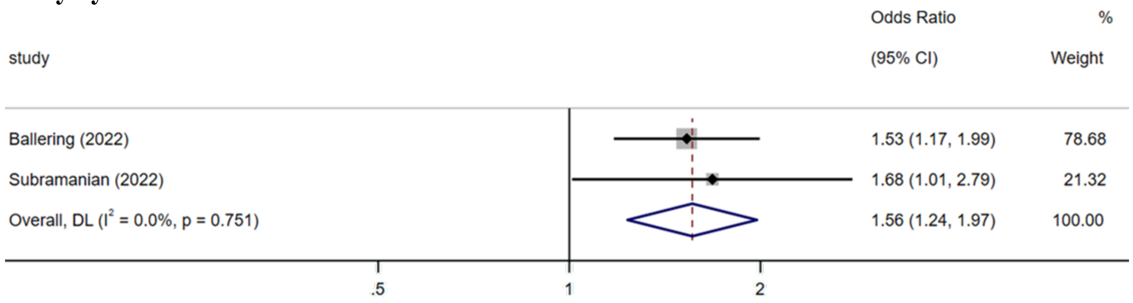

Hearing

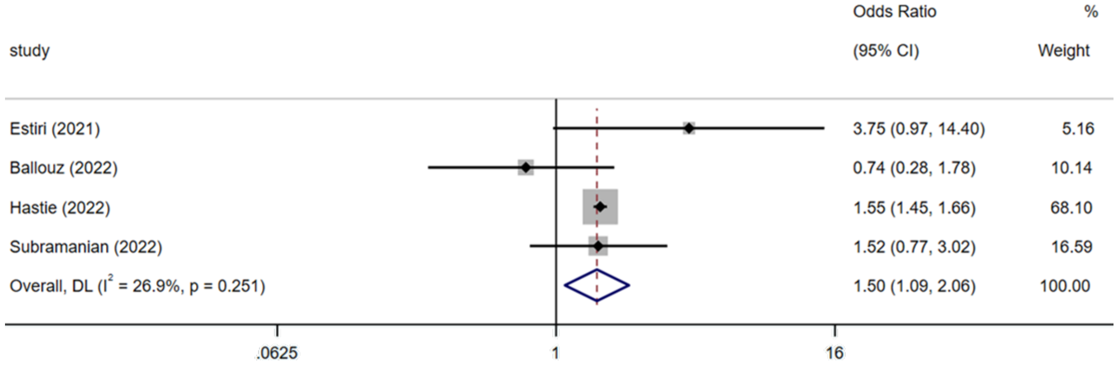

Muscle pain

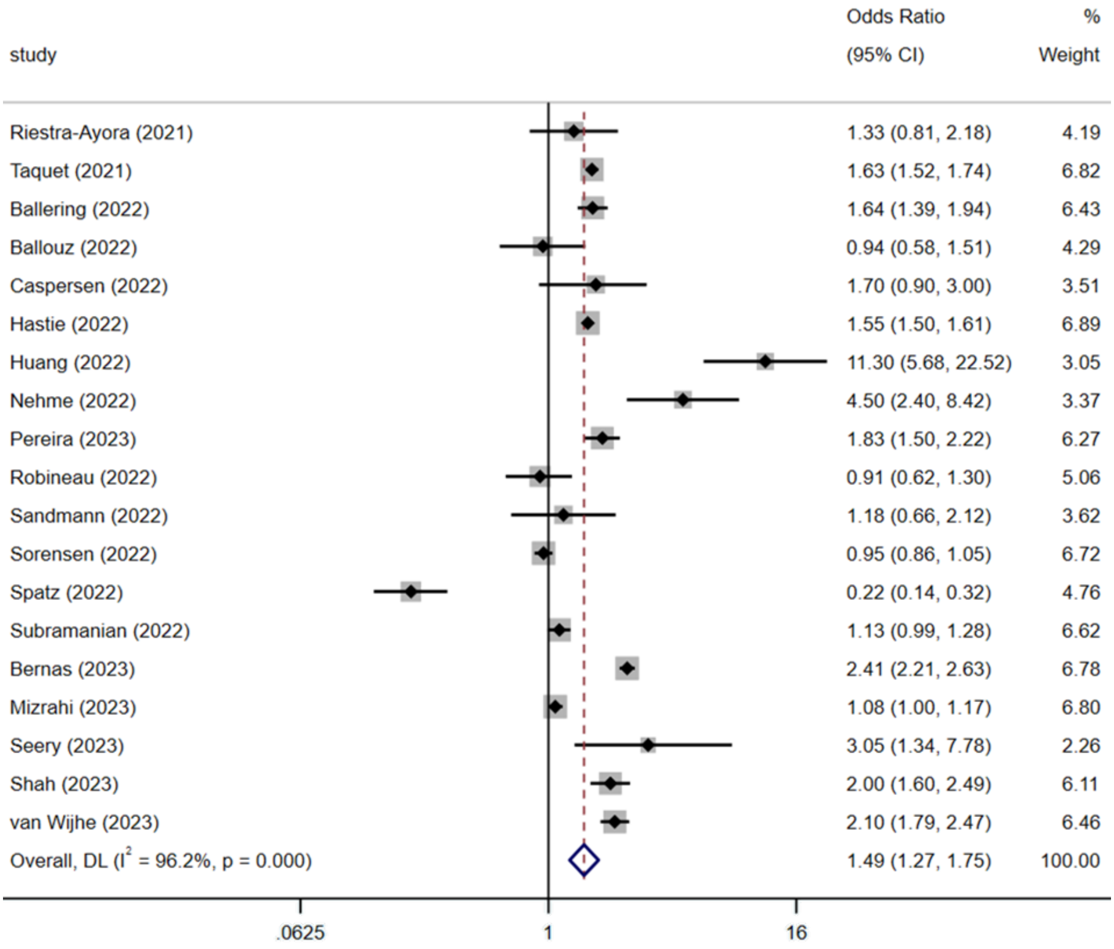

Joint pain

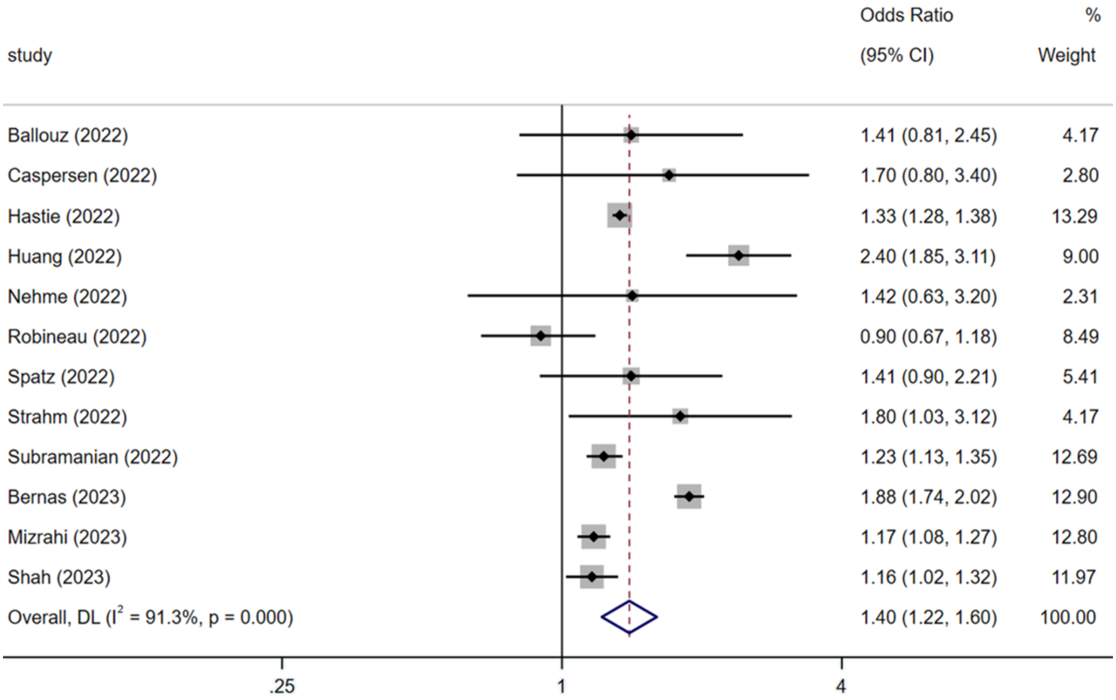

Back pain

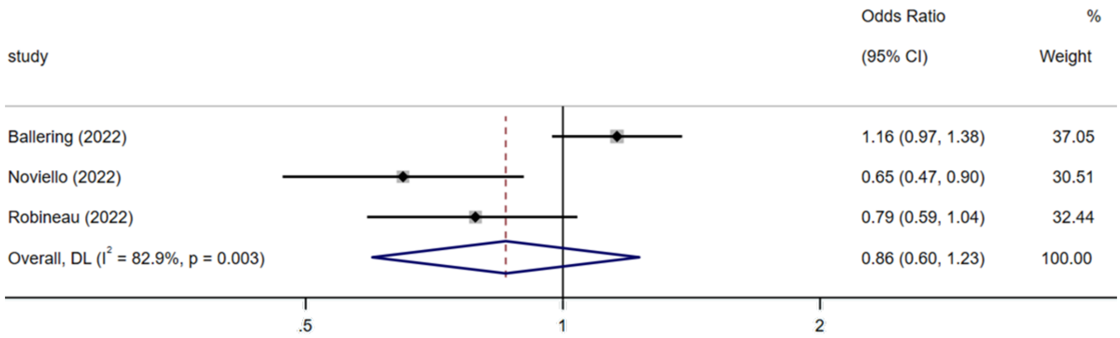

Sleep problems

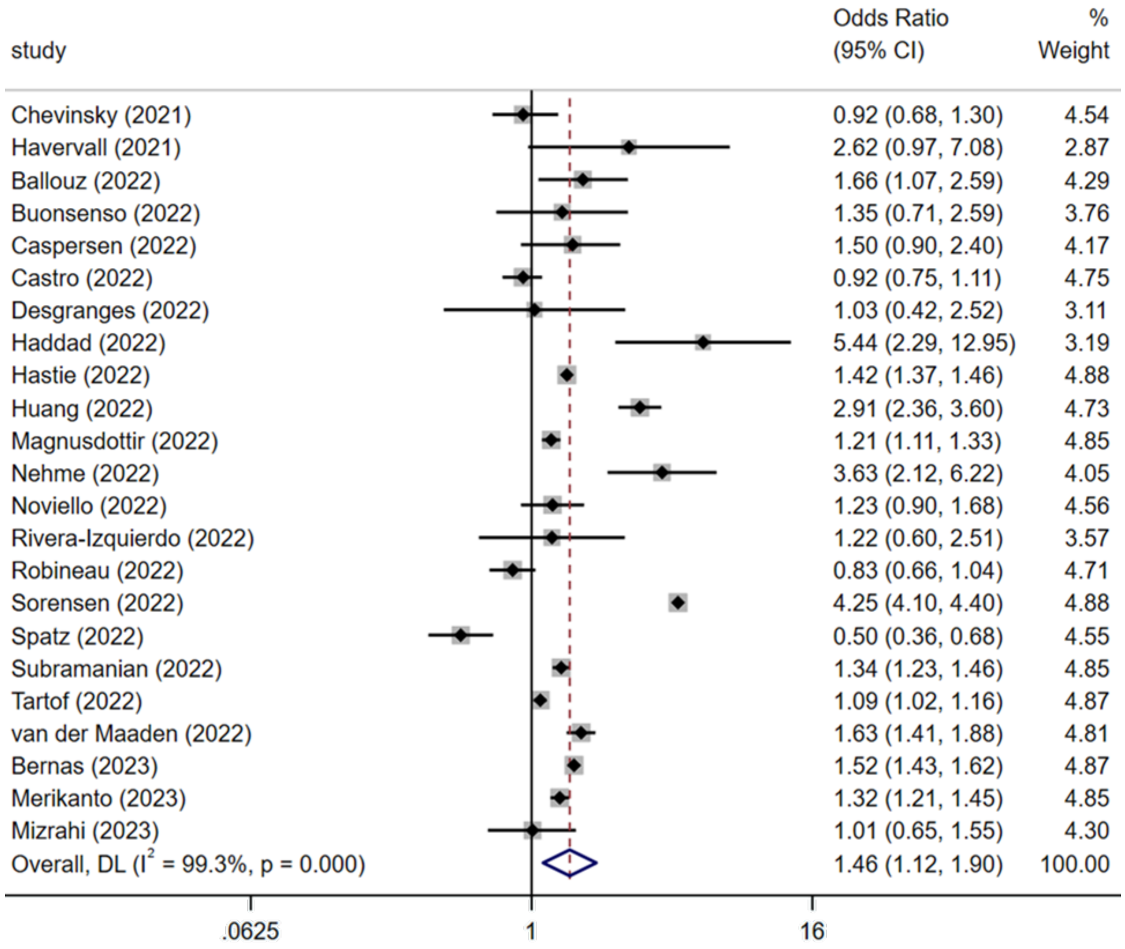

Depression

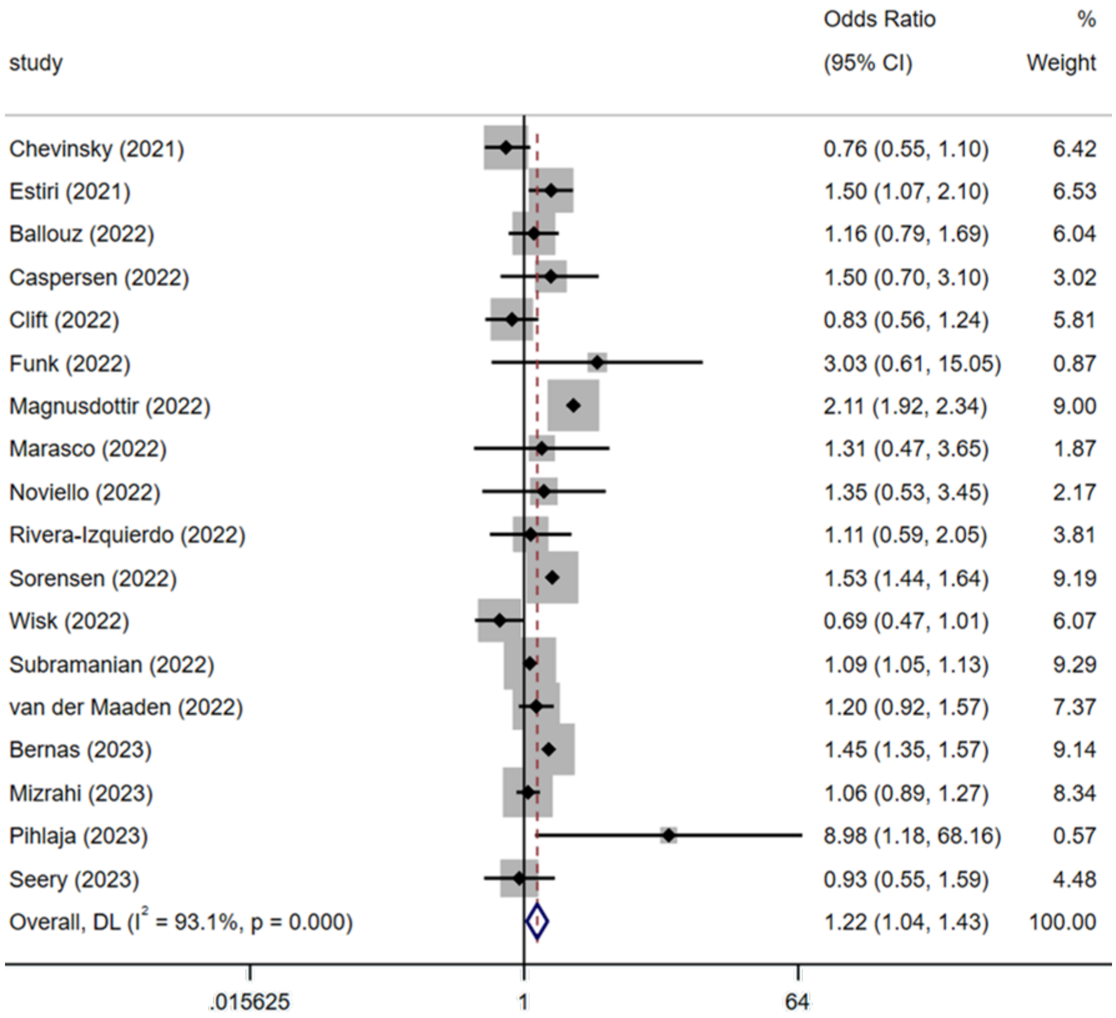
Odds Ratio
%
study
(95% CI)
Weight
Chevinsky (2021) 0.76 (0.55, 1.10) 6.42
Estiri (2021) 1.50 (1.07, 2.10) 6.53
Ballouz (2022) 1.16 (0.79, 1.69) 6.04
Caspersen (2022) 1.50 (0.70, 3.10) 3.02
Clift (2022) 0.83 (0.56, 1.24) 5.81
Funk (2022) 3.03 (0.61, 15.05) 0.87
Magnusdottir (2022) 2.11 (1.92, 2.34) 9.00
Marasco (2022) 1.31 (0.47, 3.65) 1.87
Noviello (2022) 1.35 (0.53, 3.45) 2.17
Rivera-Izquierdo (2022) 1.11 (0.59, 2.05) 3.81
Sorensen (2022) 1.53 (1.44, 1.64) 9.19
Wisk (2022) 0.69 (0.47, 1.01) 6.07
Subramanian (2022) 1.09 (1.05, 1.13) 9.29
van der Maaden (2022) 1.20 (0.92, 1.57) 7.37
Bernas (2023) 1.45 (1.35, 1.57) 9.14
Mizrahi (2023) 1.06 (0.89, 1.27) 8.34
Pihlaja (2023) 8.98 (1.18, 68.16) 0.57
Seery (2023) 0.93 (0.55, 1.59) 4.48
Overall, DL (I^2 = 93.1%, p = 0.000) 1.22 (1.04, 1.43) 100.00
.015625
1
64

Anxiety

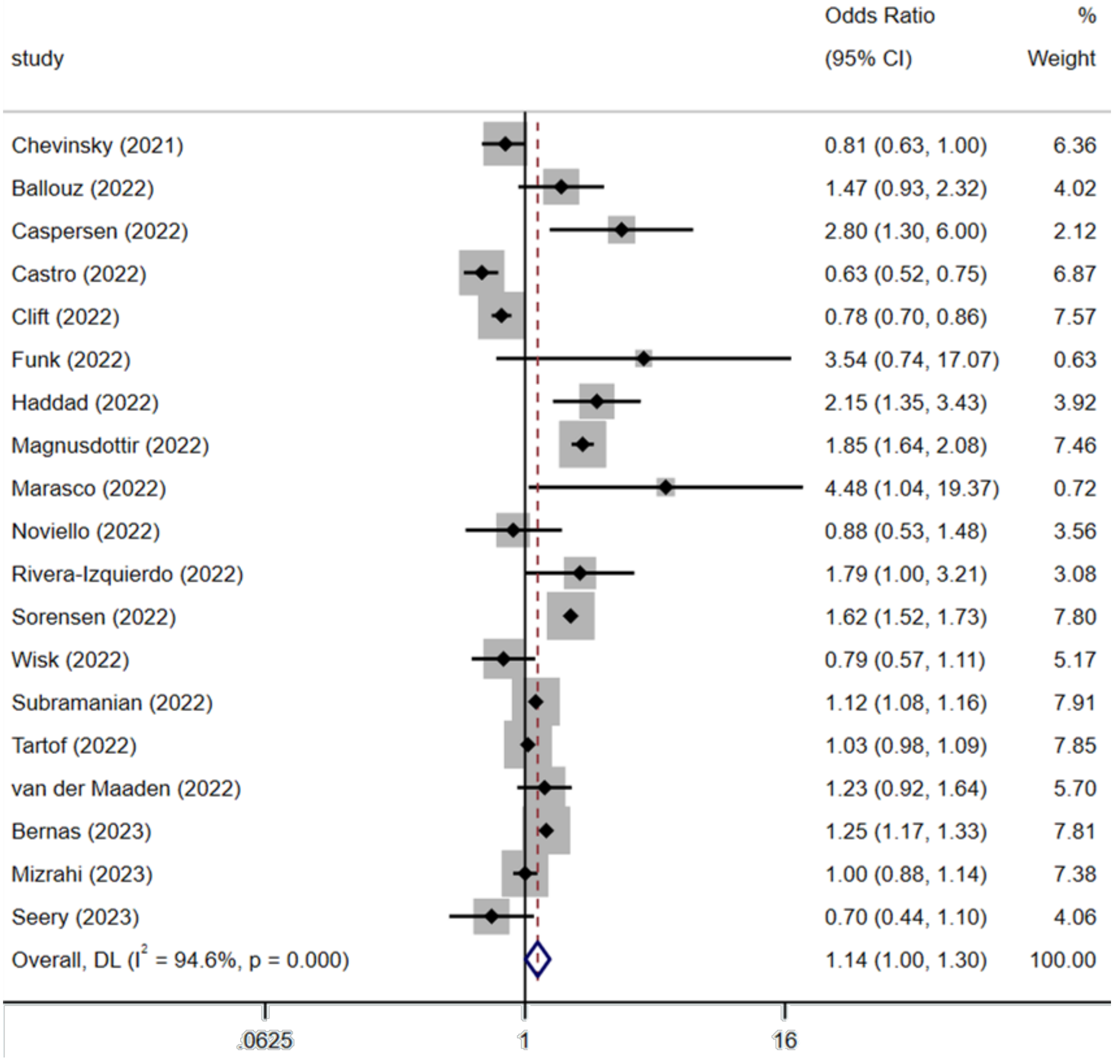
study
Odds Ratio
(95% CI)
%
Weight
Chevinsky (2021) 0.81 (0.63, 1.00) 6.36
Ballouz (2022) 1.47 (0.93, 2.32) 4.02
Caspersen (2022) 2.80 (1.30, 6.00) 2.12
Castro (2022) 0.63 (0.52, 0.75) 6.87
Clift (2022) 0.78 (0.70, 0.86) 7.57
Funk (2022) 3.54 (0.74, 17.07) 0.63
Haddad (2022) 2.15 (1.35, 3.43) 3.92
Magnusdottir (2022) 1.85 (1.64, 2.08) 7.46
Marasco (2022) 4.48 (1.04, 19.37) 0.72
Noviello (2022) 0.88 (0.53, 1.48) 3.56
Rivera-Izquierdo (2022) 1.79 (1.00, 3.21) 3.08
Sorensen (2022) 1.62 (1.52, 1.73) 7.80
Wisk (2022) 0.79 (0.57, 1.11) 5.17
Subramanian (2022) 1.12 (1.08, 1.16) 7.91
Tartof (2022) 1.03 (0.98, 1.09) 7.85
van der Maaden (2022) 1.23 (0.92, 1.64) 5.70
Bernas (2023) 1.25 (1.17, 1.33) 7.81
Mizrahi (2023) 1.00 (0.88, 1.14) 7.38
Seery (2023) 0.70 (0.44, 1.10) 4.06
Overall, DL (I² = 94.6%, p = 0.000) 1.14 (1.00, 1.30) 100.00
.0625
1
16

Gastrointestinal symptoms

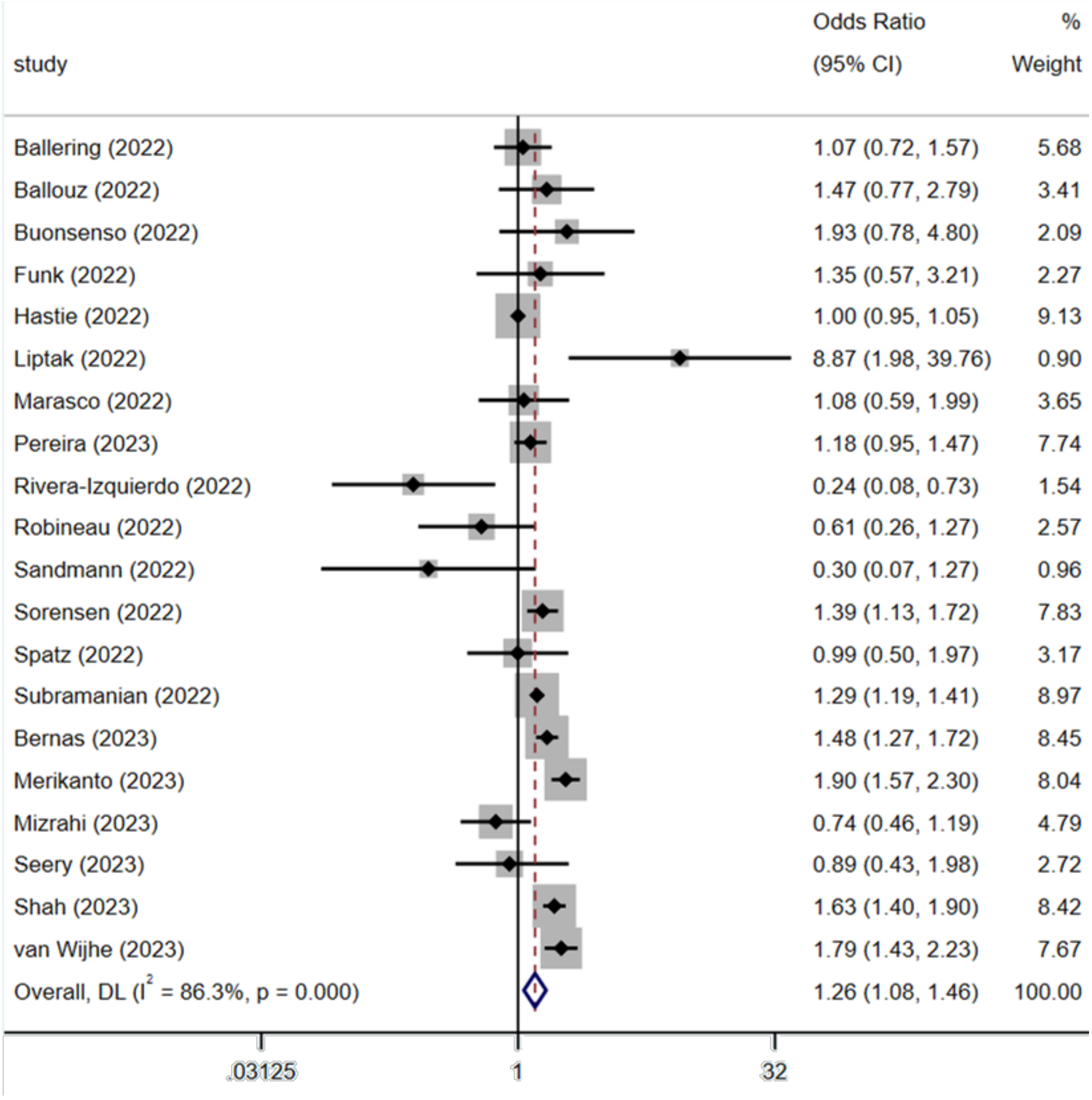

Abdominal pain

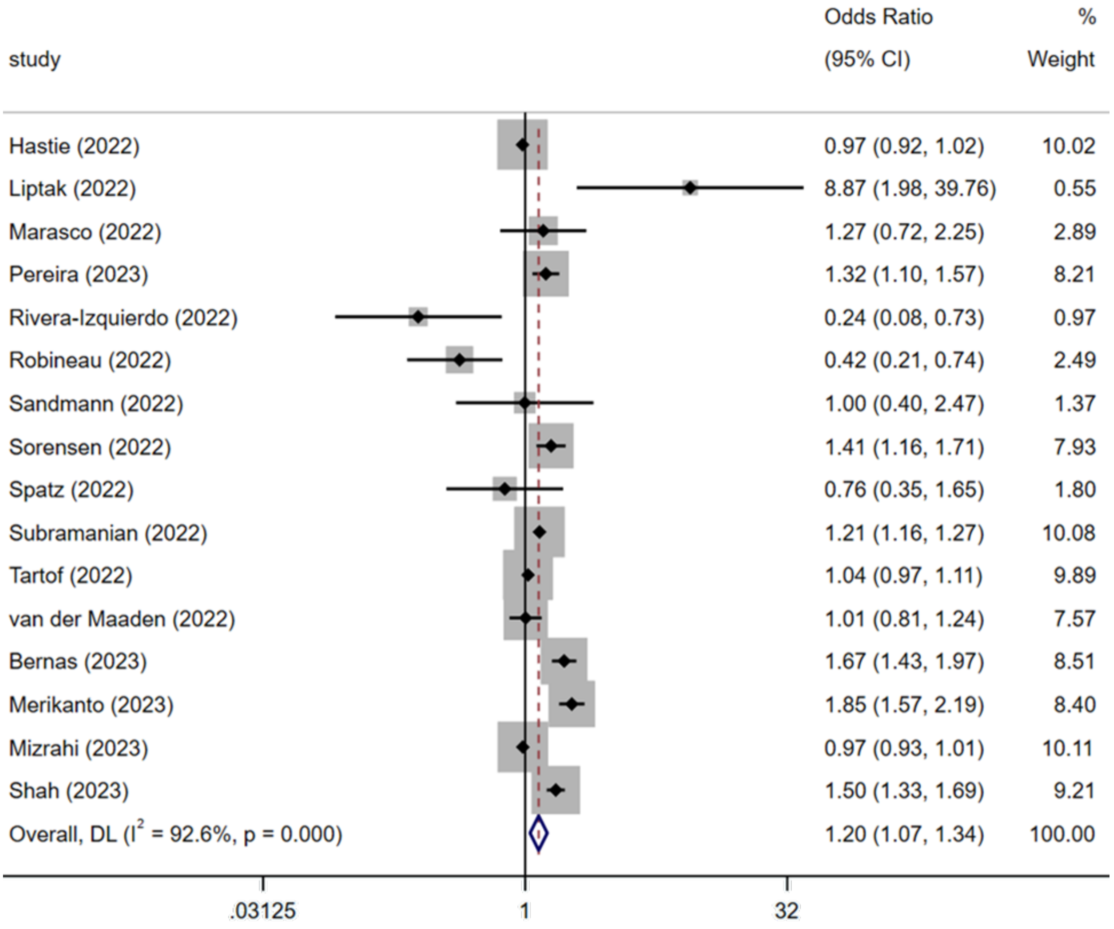

Supplementary References

1. Chevinsky JR, Tao G, Lavery AM, et al. Late conditions diagnosed 1–4 months following an initial coronavirus disease 2019 (COVID-19) encounter: a matched-cohort study using inpatient and outpatient administrative data—United States, 1 March–30 June 2020. *Clinical Infectious Diseases*. 2021;73(Supplement_1):S5-S16.
2. Estiri H, Strasser ZH, Brat GA, Semenov YR, Patel CJ, Murphy SN. Evolving phenotypes of non-hospitalized patients that indicate long COVID. *BMC medicine*. 2021;19:1-10.
3. Havervall S, Rosell A, Phillipson M, et al. Symptoms and functional impairment assessed 8 months after mild COVID-19 among health care workers. *Jama*. 2021;325(19):2015-2016.
4. Liu Y-H, Wang Y-R, Wang Q-H, et al. Post-infection cognitive impairments in a cohort of elderly patients with COVID-19. *Molecular neurodegeneration*. 2021;16:1-10.
5. Riestra-Ayora J, Yanes-Diaz J, Esteban-Sanchez J, et al. Long-term follow-up of olfactory and gustatory dysfunction in COVID-19: 6 months case–control study of health workers. *European Archives of Oto-Rhino-Laryngology*. 2021/12/01 2021;278(12):4831-4837. doi:10.1007/s00405-021-06764-y
6. Søraas A, Kalleberg KT, Dahl JA, et al. Persisting symptoms three to eight months after non-hospitalized COVID-19, a prospective cohort study. *PLoS One*. 2021;16(8):e0256142. doi:10.1371/journal.pone.0256142
7. Taquet M, Dercon Q, Luciano S, Geddes JR, Husain M, Harrison PJ. Incidence, co-occurrence, and evolution of long-COVID features: A 6-month retrospective cohort study of 273,618 survivors of COVID-19. *PLOS Medicine*. 2021;18(9):e1003773. doi:10.1371/journal.pmed.1003773
8. Al-Aly Z, Bowe B, Xie Y. Long COVID after breakthrough SARS-CoV-2 infection. *Nature medicine*. 2022;28(7):1461-1467.
9. Alessia R, Sonja R, Thomas R, et al. Low Prevalence of symptoms compatible with Long COVID in Children and Adolescents after infection with different SARS-CoV-2 variants: Results from the Ciao Corona Study. EMH SWISS MEDICAL PUBLISHERS LTD FARNSBURGERSTR 8, CH-4132 MUTTENZ, SWITZERLAND; 2022:17S-17S.
10. Ali M. Severe acute respiratory syndrome coronavirus 2 infection altered the factors associated with headache: evidence from a multicenter community-based case–control study. *Pain Reports*. 2022;7(6)
11. Ballering AV, van Zon SK, Olde Hartman TC, Rosmalen JG. Persistence of somatic symptoms after COVID-19 in the Netherlands: an observational cohort study. *The Lancet*. 2022;400(10350):452-461.
12. Ballouz T, Menges D, Anagnostopoulos A, et al. Natural course of post COVID-19 condition and implications for trial design and outcome selection: A population-based longitudinal cohort study. *medRxiv*. 2022:2022.06. 22.22276746.
13. Berg SK, Nielsen SD, Nygaard U, et al. Long COVID symptoms in SARS-CoV-2-positive adolescents and matched controls (LongCOVIDKidsDK): a national, cross-sectional study. *The lancet child & adolescent health*. 2022;6(4):240-248.
14. Berg SK, Palm P, Nygaard U, et al. Long COVID symptoms in SARS-CoV-2-positive children aged 0–14 years and matched controls in Denmark (LongCOVIDKidsDK): a national, cross-sectional study. *The Lancet Child & Adolescent Health*. 2022;6(9):614-623.
15. Bergia M, Sanchez-Marcos E, Gonzalez-Haba B, et al. Comparative study shows that 1 in 7 Spanish children with COVID-19 symptoms were still experiencing issues after 12 weeks. *Acta Paediatrica*. 2022;111(8):1573-1582.
16. Bertran M, Pereira SMP, Nugawela MD, et al. The relationship between post COVID symptoms in young people and their parents. *Journal of Infection*. 2022;85(6):702-769.
17. Bsteh G, Assar H, Gradl C, et al. Long-term outcome after COVID-19 infection in multiple sclerosis: A nation-wide multicenter matched-control study. *European Journal of Neurology*. 2022;29(10):3050-3060.

18. Buonsenso D, Munblit D, Pazukhina E, et al. Post-COVID condition in adults and children living in the same household in Italy: a prospective cohort study using the ISARIC global follow-up protocol. *Frontiers in Pediatrics*. 2022;10:447.
19. Caspersen IH, Magnus P, Trogstad L. Excess risk and clusters of symptoms after COVID-19 in a large Norwegian cohort. *European journal of epidemiology*. 2022;37(5):539-548.
20. Castro VM, Rosand J, Giacino JT, McCoy TH, Perlis RH. Case-control study of neuropsychiatric symptoms in electronic health records following COVID-19 hospitalization in 2 academic health systems. *Molecular Psychiatry*. 2022;27(9):3898-3903.
21. Clift AK, Ranger TA, Patone M, et al. Neuropsychiatric ramifications of severe COVID-19 and other severe acute respiratory infections. *JAMA psychiatry*. 2022;79(7):690-698.
22. Desgranges F, Tadini E, Munting A, et al. Post-COVID-19 syndrome in outpatients: a cohort study. *Journal of General Internal Medicine*. 2022;37(8):1943-1952.
23. Fjelltveit EB, Blomberg B, Kuwelker K, et al. Symptom burden and immune dynamics 6 to 18 months following mild severe acute Respiratory Syndrome Coronavirus 2 Infection (SARS-CoV-2): a case-control study. *Clinical Infectious Diseases*. 2023;76(3):e60-e70.
24. Funk AL, Kuppermann N, Florin TA, et al. Post–COVID-19 conditions among children 90 days after SARS-CoV-2 infection. *JAMA Network Open*. 2022;5(7):e2223253-e2223253.
25. Haddad A, Janda A, Renk H, et al. Long COVID symptoms in exposed and infected children, adolescents and their parents one year after SARS-CoV-2 infection: A prospective observational cohort study. *EBioMedicine*. 2022;84
26. Hastie CE, Lowe DJ, McAuley A, et al. Outcomes among confirmed cases and a matched comparison group in the Long-COVID in Scotland study. *Nature communications*. 2022;13(1):5663.
27. Huang L, Li X, Gu X, et al. Health outcomes in people 2 years after surviving hospitalisation with COVID-19: a longitudinal cohort study. *The Lancet Respiratory Medicine*. 2022;10(9):863-876.
28. Liptak P, Duricek M, Rosolanka R, et al. Gastrointestinal sequalae months after severe acute respiratory syndrome corona virus 2 infection: A prospective, observational study. *European journal of gastroenterology & hepatology*. 2022;34(9):925-932.
29. Magnúsdóttir I, Lovik A, Unnarsdóttir AB, et al. Acute COVID-19 severity and mental health morbidity trajectories in patient populations of six nations: an observational study. *The Lancet Public Health*. 2022;7(5):e406-e416.
30. Magnusson K, Kristoffersen DT, Dell'Isola A, et al. Post-COVID medical complaints after SARS-CoV-2 Omicron vs Delta variants-a prospective cohort study. *medRxiv*. 2022:2022.05. 23.22275445.
31. Marasco G, Cremon C, Barbaro MR, et al. Post COVID-19 irritable bowel syndrome. *Gut*. 2023;72(3):484-492.
32. Nehme M, Braillard O, Chappuis F, et al. One-year persistent symptoms and functional impairment in SARS-CoV-2 positive and negative individuals. *Journal of internal medicine*. 2022;292(1):103-115.
33. Noviello D, Costantino A, Muscatello A, et al. Functional gastrointestinal and somatoform symptoms five months after SARS-CoV-2 infection: A controlled cohort study. *Neurogastroenterology & Motility*. 2022;34(2):e14187.
34. Nugawela MD, Stephenson T, Shafran R, et al. Predictive model for long COVID in children 3 months after a SARS-CoV-2 PCR test. *BMC Medicine*. 2022/11/30 2022;20(1):465. doi:10.1186/s12916-022-02664-y
35. Stephenson T, Pinto Pereira SM, Shafran R, et al. Physical and mental health 3 months after SARS-CoV-2 infection (long COVID) among adolescents in England (CLoCk): a national matched cohort study. *The Lancet Child & Adolescent Health*. 2022;6(4):230-239. doi:10.1016/S2352-4642(22)00022-0

36. Snehal MPP, Manjula DN, Natalia KR, et al. Post-COVID-19 condition at 6 months and COVID-19 vaccination in non-hospitalised children and young people. *Archives of Disease in Childhood*. 2023;108(4):289. doi:10.1136/archdischild-2022-324656
37. Rivera-Izquierdo M, Láinez-Ramos-Bossini AJ, de Alba IG-F, et al. Long COVID 12 months after discharge: persistent symptoms in patients hospitalised due to COVID-19 and patients hospitalised due to other causes—a multicentre cohort study. *BMC Medicine*. 2022/02/23 2022;20(1):92. doi:10.1186/s12916-022-02292-6
38. Robineau O, Wiernik E, Lemogne C, et al. Persistent symptoms after the first wave of COVID-19 in relation to SARS-CoV-2 serology and experience of acute symptoms: A nested survey in a population-based cohort. *The Lancet Regional Health – Europe*. 2022;17doi:10.1016/j.lanepe.2022.100363
39. Sandmann FG, Tessier E, Lacy J, et al. Long-Term Health-Related Quality of Life in Non-Hospitalized Coronavirus Disease 2019 (COVID-19) Cases With Confirmed Severe Acute Respiratory Syndrome Coronavirus 2 (SARS-CoV-2) Infection in England: Longitudinal Analysis and Cross-Sectional Comparison With Controls. *Clin Infect Dis*. Aug 24 2022;75(1):e962-e973. doi:10.1093/cid/ciac151
40. Sørensen AIV, Spiliopoulos L, Bager P, et al. A nationwide questionnaire study of post-acute symptoms and health problems after SARS-CoV-2 infection in Denmark. *Nature Communications*. 2022/07/21 2022;13(1):4213. doi:10.1038/s41467-022-31897-x
41. Spiliopoulos L, Sørensen AIV, Bager P, et al. Post-acute symptoms four months after SARS-CoV-2 infection during the Omicron period: a nationwide Danish questionnaire study. medRxiv; 2022.
42. Spatz ES, Gottlieb M, Wisk LE, et al. Three-Month Symptom Profiles Among Symptomatic Adults With Positive and Negative Severe Acute Respiratory Syndrome Coronavirus 2 Tests: A Prospective Cohort Study From the INSPIRE Group. *Clinical infectious diseases : an official publication of the Infectious Diseases Society of America*. 2023/05// 2023;76(9):1559-1566. doi:10.1093/cid/ciac966
43. Wisk LE, Gottlieb MA, Spatz ES, et al. Association of Initial SARS-CoV-2 Test Positivity With Patient-Reported Well-being 3 Months After a Symptomatic Illness. *JAMA Network Open*. 2022;5(12):e2244486-e2244486. doi:10.1001/jamanetworkopen.2022.44486
44. Strahm C, Seneghini M, Güsewell S, et al. Symptoms Compatible With Long Coronavirus Disease (COVID) in Healthcare Workers With and Without Severe Acute Respiratory Syndrome Coronavirus 2 (SARS-CoV-2) Infection—Results of a Prospective Multicenter Cohort. *Clinical Infectious Diseases*. 2022;75(1):e1011-e1019. doi:10.1093/cid/ciac054
45. Subramanian A, Nirantharakumar K, Hughes S, et al. Symptoms and risk factors for long COVID in non-hospitalized adults. *Nature Medicine*. 2022/08/01 2022;28(8):1706-1714. doi:10.1038/s41591-022-01909-w
46. Tartof SY, Malden DE, Liu I-LA, et al. Health Care Utilization in the 6 Months Following SARS-CoV-2 Infection. *JAMA Network Open*. 2022;5(8):e2225657-e2225657. doi:10.1001/jamanetworkopen.2022.25657
47. van der Maaden T, Mutubuki EN, de Bruijn S, et al. Prevalence and Severity of Symptoms 3 Months After Infection With SARS-CoV-2 Compared to Test-Negative and Population Controls in the Netherlands. *The Journal of Infectious Diseases*. 2023;227(9):1059-1067. doi:10.1093/infdis/jiac474
48. Bernas SN, Baldauf H, Real R, et al. Post-COVID-19 condition in the German working population: A cross-sectional study of 200,000 registered stem cell donors. *Journal of Internal Medicine*. 2023;293(3):354-370.
49. Merikanto I, Dauvilliers Y, Chung F, et al. Sleep symptoms are essential features of long-COVID–Comparing healthy controls with COVID-19 cases of different severity in the international COVID sleep study (ICOSS-II). *Journal of Sleep Research*. 2023;32(1):e13754.
50. Miller MJ, Feldstein LR, Holbrook J, et al. Post-COVID conditions and healthcare utilization among adults with and without disabilities—2021 Porter Novelli FallStyles survey. *Disability and health journal*. 2023;16(2):101436.

51. Mizrahi B, Sudry T, Flaks-Manov N, et al. Long covid outcomes at one year after mild SARS-CoV-2 infection: nationwide cohort study. *Bmj*. 2023;380
52. Pihlaja RE, Kauhanen L-LS, Ollila HS, et al. Associations of subjective and objective cognitive functioning after COVID-19: A six-month follow-up of ICU, ward, and home-isolated patients. *Brain, Behavior, & Immunity - Health*. 2023/02/01/ 2023;27:100587. doi:https://doi.org/10.1016/j.bbih.2023.100587
53. Seery V, Raiden S, Penedo JMG, et al. Persistent symptoms after COVID-19 in children and adolescents from Argentina. *International Journal of Infectious Diseases*. 2023/04/01/ 2023;129:49-56. doi:https://doi.org/10.1016/j.ijid.2023.01.031
54. Shah A, Subramanian A, Lewis J, et al. Long Covid symptoms and diagnosis in primary care: a cohort study using structured and unstructured data in The Health Improvement Network primary care database. medRxiv; 2023.
55. van Wijhe M, Fogh K, Ethelberg S, et al. Persistent Symptoms and Sequelae After Severe Acute Respiratory Syndrome Coronavirus 2 Infection Not Requiring Hospitalization: Results From Testing Denmark, a Danish Cross-sectional Survey. *Open Forum Infectious Diseases*. 2023;10(1):ofac679. doi:10.1093/ofid/ofac679
